# Comparative Analysis of the Systematics and Evolution of the *Pampus* Genus of Fish (Perciformes: Stromateidae) Based on Osteology, Population Genetics and Complete Mitogenomes

**DOI:** 10.3390/ani14050814

**Published:** 2024-03-06

**Authors:** Cheng Zhang, Hanjing Liu, Xiang Huang, Zi Yuan, Shun Zhang, Shanliang Xu, Jing Liu, Yajun Wang, Danli Wang, Jiabao Hu

**Affiliations:** 1 School of Marine Science, Ningbo University, Ningbo 315211, China; 2 School of Life Sciences, Sun Yat-sen University, Guangzhou 510275, China; 3 Laboratory of Marine Organism Taxonomy and Phylogeny, Qingdao Key Laboratory of Marine Biodiversity and Conservation, and The Key Laboratory of Experimental Marine Biology, Centre for Ocean Mega-Science, Institute of Oceanology, Chinese Academy of Sciences, Qingdao 266071, China; 4 Key Laboratory of Applied Marine Biotechnology (Ningbo University), Ministry of Education, Ningbo 315211, China; 5 Key Laboratory of Marine Biotechnology of Zhejiang Province, Ningbo University, Ningbo 315211, China

**Keywords:** *Pampus*, osteology, population genetics, mitogenome, species delimitation, evolutionary history

## Abstract

**Simple Summary:**

*Pampus* is a genus of fish of commercial importance in Asia. This is a comprehensive study on the species delimitation and evolution history of *Pampus* species. This study integrated information on the skeletal structure and mitochondrial and nuclear molecular data of the genus *Pampus* to define their species delimitation. Based on these findings, we suggest that *P. argenteus* and *P. echinogaster* should be classified as the same species and *P. liuorum* is speculated to be a valid species. *P. cinereus* is closely related to *P. minor*, *P. chinensis* and *P. punctatissimus*, but these are different species. *Pampus* can be divided into six species: *P. argenteus*, *P. punctatissimus*, *P. cinereus*, *P. chinensis*, *P. minor*, and *P. liuorum*. The aim of this study was to resolve the controversies about the phylogeny and taxonomy of *Pampus* and provide a robust delimitation of *Pampus* for fisheries management.

**Abstract:**

*Pampus* is a widespread species of fish in the western Pacific and Indian Oceans that has significant commercial worth. Its evolutionary history and phylogenetics are still poorly understood, and details on its intraspecific taxonomy are debatable, despite some morphological and molecular research. Here, we analyzed this species using skeletal structure data as well as nuclear (*S7* gene) and mitochondrial genetic information (*COI*, *D-loop* and mitogenomes). We found that the genetic distance between *P. argenteus* and *P. echinogaster* was much smaller than that between other *Pampus* species, and both maximum likelihood and Bayesian phylogenetic trees yielded almost identical tree topologies. An additional and adjacent M repeat was found in the downstream region of the IQM gene cluster of *P. argenteus* and *P. echinogaster*, and the *trnL2* gene of *P. minor* was translocated. The genus *Pampus* experienced early rapid radiation during the Palaeocene with major lineages diversifying within a relatively narrow timescale. Additionally, three different methods were conducted to distinguish the genus *Pampus* species, proving that *P. argenteus* and *P. echinogaster* are the same species, and *P. liuorum* is speculated to be a valid species. Overall, our study provides new insights not only into the evolutionary history of *Pampus* but its intraspecific taxonomy as well.

## 1. Introduction

*Pampus* fishes belong to the family Stromateidae and the order Perciformes and have high nutritional and economic values as a globally distributed species of fish [1,2]. In the 1905s, Fowler (1905) formally proposed that *Stromateoides* and *Stromateus* should be replaced by *Pampus* as the genus name, a view that has been widely accepted and remains in use today [3,4,5]. However, the taxonomic relationship of *Pampus* species has been a subject of controversy since the genus was established and has yet to be fully resolved [4,5,6,7].

At the beginning of these studies, the species delimitation of the genus *Pampus* was primarily based on morphological characteristics. For example, Regan (1920) divided *Pampus* into two species based on the characteristics of their caudal fins: *P. chinensis* had a truncated or shallowly forked caudal fin, while *P. cinereus* had a deeply forked caudal fin with an extended lower lobe [8]. Wang (1958) also separated *Pampus* into two species, *P. argenteus* and *P. chinensis*, based on the morphological characteristics of their fins, and considered *P. cinereus* to be synonymous with *P. argenteus* [9]. Liu divided *Pampus* in the coastal regions of China into five species and introduced a new species, *P. minor* [10,11]. Simultaneously, a new species of *Pampus* was also reported, namely *P. liuorum*, which is distributed in the southeast coastal regions of China, and the fish was thought to be the sixth species of *Pampus*, but its validity is doubtful [4,5]. Wei et al. (2022) believed that *P. candidus* was a synonymous species of *P. echinogaster* [12]. Jawad and Jig (2017) classified *Pampus* into eight species based on seven skeletal characteristics of the axial skeleton [1]. Due to the high similarity of morphological characteristics among *Pampus* species, the taxonomy of the genus has been complex and confusing. In recent decades, with the rapid development of molecular biotechnology, molecular marker technology has been widely used for species identification [13,14]. For instance, Divya et al. (2017) proposed that the genus *Pampus* can be divided into seven valid species based on the *COI* gene, including *Pampus sp1*, *Pampus sp2*, *P. cinereus/P.nozawae*, *P. chinensis*, *P. punctatissimus*, *P. minor*, and *Pampus* sp., consisting of *P. echinogaster* and *P. argenteus* [6]. Similarly, Li et al. (2019) also identified seven valid species in the genus *Pampus*, including the new species *P. liuorum* [5]. However, the use of the same molecular markers to analyze the species delimitation of *Pampus* led to varying conclusions. This may be due to misidentification of *Pampus* by some researchers. For example, *P. argenteus* in Indian waters was found to be clearly different from *P. argenteus* in the South China Sea [6]. The fish collected from Malaysia and labelled *P. argenteus* should actually be *P. cinereus*, and *P. minor* and *P. cinereus* were incorrectly identified as *P. argenteus* in the GenBank data [7]. These inaccuracies have further confused the taxonomic delimitation of the genus *Pampus*. At present, the latest global reports indicate that there are seven effective species in this genus, including *P. argenteus*, *P. minor*, *P. punctatissimus*, *P. chinensis*, *P. cinereus*, *P. candidus*, and *Pampus* sp [15]. However, the lack of taxonomic publications and reference data has hindered the precise species delimitation and evolutionary research of the genus *Pampus*. Using only a few genes or gene fragments may not yield the most convincing conclusions [16]. The study of multiple genes combinations and even the whole mitochondrial genome has become a common practice to obtain more reliable results [17,18].

Hence, we aim to resolve the taxonomic issues surrounding *Pampus* by utilizing comprehensive delimitation methods. In this study, we collected samples of *Pampus*, described the major skeletal system of adult fish, analyzed the key features of their adult skeletal systems, and utilized both mitochondrial and nuclear molecular markers and complete mitochondrial genomes to clarify the taxonomic classification and evolutionary history of the genus *Pampus*.

## 2. Materials and Methods

### 2.1. Sample Collection and PCR Amplification

From November to December 2021, commercial trawlers were used by fishermen to collect *Pampus* specimens from coastal regions of China. All the samples were identified by J. Liu based on previous taxonomic works [10,11,19,20,21], including *P. argenteus* (N = 40), *P. punctatissimus* (N = 36), *P. cinereus* (N = 36), *P. chinensis* (N = 34), *P. echinogaster* (N = 35), *P. minor* (N = 36), and *P. liuorum* (N = 1). Fresh muscle tissue samples were taken and preserved in 95% ethanol. Total genomic DNA was extracted using the traditional phenol–chloroform method [22]. The high-quality DNA was diluted to 100 ng/µL and stored in 1.5 mL centrifuge tube (Guangzhou Jet Bio-Filtration Co., Ltd., Guangzhou, China) at −20 °C. The primers for the *COI* gene were F: 5′-GCATGAGCTGGTATAGTAGG-3′ and R: 5′-GCTCAGACCATGCCCATATATC-3′, the primers for the *D-loop* gene were F: 5′-ACCATCCAGCTCATATCTTAATG-3′ and R: 5′-GAATGATAGCTATGTCACGAG-3′, and the primers for nuclear *S7* gene were F: 5′-TGGCCTCTTCCTTGGCCGTC-3′ and R: 5′-AACTCGTCTGGCTTTTCGCC-3′. The PCR condition was performed with 12.5 μL 2× Taq PCR MasterMix, 2 μL DNA, 2 μL of F/R primers, and 6.5 μL DNase-Free deionized water. The PCR amplification conditions were as follows: initial denaturation for 3 min at 94 °C, 35 cycles of 30 s at 94 °C for denaturation, 30 s at 54 °C for annealing, 1 min at 72 °C for extension, and a final extension at 72 °C for 10 min. The purified PCR products were sent to Sanger Biotech Co., Ltd. (Shanghai, China) for sequencing and deposition.

### 2.2. Preparation of Bone Specimens

Six different species of adult *Pampus* fish, including *P. argenteus*, *P. punctatissimus*, *P. cinereus*, *P. chinensis*, *P. echinogaster*, and *P. minor*, were chosen for this study. The skeleton specimens were prepared using the method outlined by Su et al. (2012) [23]. However, the skeletal information of *P. liuorum* has not yet been collected.

### 2.3. Mitogenome Sequencing, Assembly and Annotation

High-throughput sequencing libraries were constructed using the Illumina TruSeq DNA PCR-Free HT Kit and sequenced on an Illumina HiSeq 2500 with the 250 bp paired-end strategy. The quality of the raw sequencing reads was assessed using FastQC version 0.11.9, and the adapter sequences and low-quality reads were removed using Trimmomatic version 0.39. The filtered sequencing reads were then assembled into a complete mitogenome using NOVOPlasty 4.2. The tRNAs’ typical clover-leaf secondary structure and anticodon were identified using tRNAscan-SE 2.0. Finally, the codon usage of protein-coding genes (PCGs) and the nucleotide composition of the mitogenomes were determined using MEGA 5.0.

### 2.4. Phenotypic Analysis of Bone Specimens

After the preparation of *Pampus* bone specimens, the morphological and structural differences of their bones were observed using a stereomicroscope. We collected and documented bone specimen information for the six *Pampus* species (namely, *P. argenteus*, *P. punctatissimus*, *P. cinereus*, *P. chinensis*, *P. echinogaster*, and *P. minor*).

### 2.5. Population Structure Analysis

The *COI*, *D-loop* and *S7* gene datasets were analyzed independently and aligned using the ClustalW multiple-alignment program as implemented in BioEdit v7.1.9 software [24] and checked manually for misalignments. The mismatch and neutrality tests were performed using DNAsp v5.10.01. The genetic distances among *Pampus* populations were analyzed using MEGA version 5.0. The best-fit models of nucleotide substitution were selected using jModeltest v2 [25] based on the Akaike information criterion (AIC) value for Bayesian inference (BI), with GTR+I+G as the best-fit evolutionary models. BI analysis was carried out using four simultaneous Markov chain Monte Carlo (MCMC) for 5,000,000 generations, sampled every 1000 generations by MrBayes 3.2.7a [26]. The average standard deviation of split frequencies was less than 0.01. The topology tree and the Bayesian posterior probabilities were derived after excluding the first 25% of “burn-in” trees. The maximum likelihood (ML) trees were constructed with RAxML [27] using GTR+GAMMA as the best-fit evolutionary models, and the analyses were performed with 1000 bootstrap replicates to calculate the node support values. PAUP v4.0a167 [28] was used to conduct maximum parsimony (MP) tree analysis through heuristic parsimony research, in which the minimum number of evolutionary steps was needed. The initial tree was obtained by step-by-step addition, and every 100 sequences were randomly added. The branch swapping algorithm employed was tree bisection and reconnection. All data were considered unordered and unweighted. The confidence values for the branches of the obtained system tree were represented by 1000 bootstrap replicates, and other evaluation factors including tree length (TL), CI value (consistency index), RI value (retention index), and RC value (rescaled consistency index) were also calculated. Finally, the phylogenetic trees were constructed using FigTree v1.4.3.

### 2.6. Codon Analysis

The relative synonymous codon usage (RSCU) was calculated using CodonW v1.4.2. The ΔRSCU method was applied to identify optimal codons, which were defined as those with a ΔRSCU value > 0.08 and an RSCU value greater than 1 in the high sample group and less than 1 in the low sample group. The RSCU value of all codons, except for AUG, UGG, and three stop codons (TAA, TAG, and TGA), was calculated. The resulting RSCU values for the remaining 59 codons were employed for cluster analysis using SPSS22.0.

### 2.7. Phylogenetic Analysis and Divergence Time Analysis

In this study, all known mitogenomes of Scombriformes from the NCBI database were collected, and the complete or nearly complete mitogenomes of the 92 fish species from 17 families were used for phylogenetic analysis, with *Sillago asiatica* (NC_025337.1) being selected as the outgroup. ML analysis was performed using IQ-TREE with 10,000 ultrafast bootstrap replicates and partition models to evaluate branches. BI was conducted using MrBayes 3.2.7a [26], using four MCMC chains running for 10,000,000 generations with a sample frequency of 1000. The software Tracer v1.7.1 was used to ensure convergence by diagnosing the effective sample size values of all sampled parameters [29]. The phylogenetic trees and node labels were visualized using Tree v1.4.3. Divergence times of major clades were performed using BEAST v2.5.0 with relaxed uncorrelated lognormal clocks, random starting trees, and the Yule speciation model [30]. Posterior distributions of parameters, including the tree, were approximated by sampling from two independent MCMC analyses. Partition of data and model selection were set as before. Samples from the posterior were drawn every 1000 steps over a total of 50,000,000 steps per MCMC run, following a discarded burn-in of 50% steps. The resulting distributions were combined and verified using Tracer v1.7.1. The maximum clade credibility tree topology was identified using TreeAnnotator v2.7.0 with a burn-in of 50% and mean node heights calculated from the posterior distribution of trees. In addition, the differentiation times between *Peprilus burti* and *Peprilus triacanthus* (1.0–1.9 Mya) [31] and between *Seriolella porosa* and *Psenopsis anomala* (5.3–32.8 Mya) [32] were used for time calibration.

### 2.8. Species Delimitation Analysis

Three different methods were used to evaluate the taxonomic units from DNA identification of the mitogenome dataset. These methods include automatic barcode gap discovery (ABGD) [33], assemble species by automatic partitioning analysis (ASAP) [34], and the Poisson tree process (PTP) model [35]. The ABGD method, which is a useful tool for distinguishing species based on the aligned sequence sets, was applied to the mitogenome alignments using default parameters via an online tool (https://bioinfo.mnhn.fr/abi/public/abgd/abgdweb.html (accessed on 7 March 2023)). The ASAP method, which is also a K2P distance-based approach, considered the partition with the smallest score as the final outcome for species delimitation. Finally, the PTP method, which is a tree-based method for species delimitation, uses aggregation theory to examine species-level processes.

## 3. Results

### 3.1. Morphological Analyses

**Figure 1:**
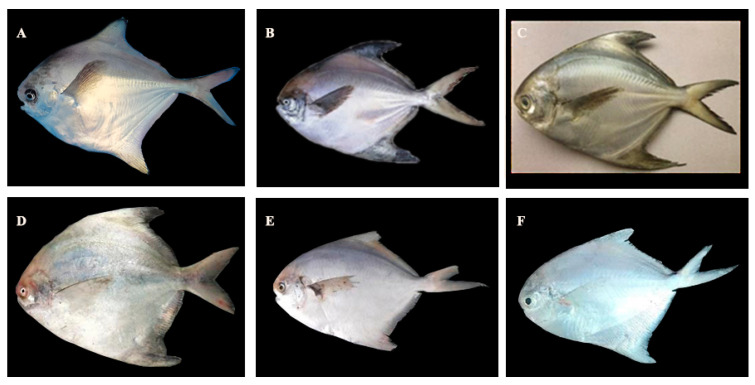
Photographs of the six *Pampus* species of this study. Note: (**A**). *Pampus argenteus*, (**B**). *Pampus punctatissimus*, (**C**). *Pampus cinereus*, (**D**). *Pampus chinensis*, (**E**). *Pampus echinogaster*, (**F**). *Pampus minor*.

**Figure 2:**
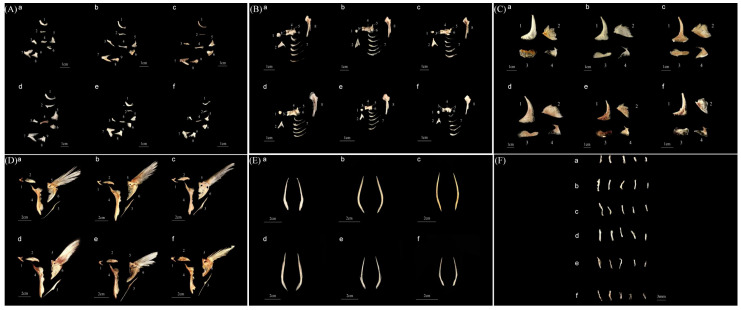
Morphological description of bone structures of the genus *Pampus*. Note: a. *Pampus argenteus*, b. *Pampus punctatissimus*, c. *Pampus cinereus*, d. *Pampus chinensis*, e. *Pampus echinogaster*, f. *Pampus minor*. (**A**) the mandibular arch: 1. maxilla, 2. premaxilla, 3. palatine bone, 4. mesopterygoid bone, 5. metapterygoid bone, 6. quadrate bone, 7. dentary bone, 8. articular bone, 9. angular bone; (**B**) the hyoid arch: 1. basihyal bone, 2. urohyal bone, 3. hypohyal bone, 4. ceratohyal bone, 5. interhyal bone, 6. epihyal bone, 7. branchiostegal ray, 8. hyomandibular bone; (**C**) the opercular series: 1. preopercular bone, 2. opercular bone, 3. interopercular bone, 4. subopercular bone; (**D**) the pectoral girdle: 1. posttemporal, 2. supracleithrum, 3. postcleithrum, 4. cleithrum, 5. scapula, 6. coracoid; (**E**) the pelvic girdle; (**F**) the periorbital bone.

In this study, the photographs of *P. argenteus*, *P. punctatissimus*, *P. cinereus*, *P. chinensis*, *P. echinogaster*, and *P. minor* were taken, as shown in Figure 1. The parameters of the body length and weight of *P. argenteus* and *P. echinogaster* were relatively close, which were between 130–134 mm and 78–80 g, respectively. The parameters of the body length and body weight of *P. chinensis* were relatively the largest, which were 198.13 ± 10.69 mm and 315.47 ± 30.05 g, respectively, while those of *Pampus minor* were relatively the smallest, which were 107.10 ± 10.86 mm and 59.63 ± 4.94 g, respectively (Table 1). *P. argenteus*, *P. echinogaster*, and *P. cinereus* had a relatively large number of vertebrae, ranging from 38 to 40, while *P. chinensis* and *P. minor* had fewer vertebrae, with only 30–32, and *P. punctatissimus* had a total number of vertebrae between them, with only 34–35. Additionally, the number of dorsal ribs was counted, revealing that *P. cinereus* had the largest number of dorsal ribs, with 26, while *P. argenteus* and *P. echinogaster* both had 24, and the remaining three *Pampus* species had relatively small numbers, with only 15–19. The number of abdominal ribs was relatively similar among the six *Pampus* species, ranging from 11 to 14. In terms of dorsal fin rays, *P. argenteus* and *P. echinogaster* had the largest number, ranging from 57 to 60, while *P. chinensis*, *P. punctatissimus*, and *P. minor* had similar numbers, ranging from 49 to 55. *P. cinereus* had the smallest number of dorsal fin rays, with 42. The skeletal structure of the six *Pampus* fishes can be classified into two types based on their occurrence process: membrane bone and cartilage bone. The mandibular arch’s skeletal structure includes the maxilla, premaxilla, palatine bone, mesopterygoid bone, metapterygoid bone, and quadrate bone, which all belong to the skeletal properties of the membrane bone. The dentary bone, articular bone, and angular bone in the skeletal structure of the mandibular arch belong to the skeletal properties of the cartilage bone. Additionally, the hyoid arch contains several bones on each side, including the hyomandibular bone, basihyal bone, hypohyal bone, ceratohyal bone, interhyal bone, and epihyal bone. The opercular series includes the branchiostegal ray, preopercular bone, opercular bone, interopercular bone, and subopercular bone. The girdle bone of the pectoral fin is called the pectoral girdle, which consists of the posttemporal, supracleithrum, cleithrum, scapula, coracoid, and postcleithrum (Figure 2). The detailed skeletal information of *P. argenteus*, *P. punctatissimus*, *P. cinereus*, *P. chinensis*, *P. echinogaster*, and *P. minor* is shown in Table 2.

### 3.2. Molecular Marker Analysis

The evolutionary dynamics of biological populations were detected using two methods: neutral tests and mismatch analyses. If at least one of the test values of Tajima’s D test and Fu’s Fs test was negative and significantly deviated from neutral [36,37], and the observed values of mismatch analyses showed an approximate unimodal distribution, it indicated that the population has experienced an expansion event. In this study, we successfully amplified and sequenced the 664 bp *COI* gene from 217 individuals across six *Pampus* populations. The results of neutral tests and mismatch analyses are presented in Table 3 and Figure S1, the Tajima’s D test value of the *P. cinereus* population was positive, and the Fu’s Fs values of *P. argenteus* and *P. echinogaster* were negative, and only the *P. argenteus* population exhibited significant differences. Mismatch analysis results revealed that the *P. argenteus* and *P. echinogaster* populations were unimodal distributions of pairwise differences, whereas the other populations were not. Regarding interspecies analysis, the genetic distance between *P. argenteus* and *P. echinogaster* was the lowest (0.00251), while that between *P. argenteus* and *P. minor* was the largest (0.12598). The genetic distances between *P. cinereus* and *P. punctatissimus* or *P. chinensis* and between *P. chinensis* and *P. punctatissimus* ranged from 0.04726 to 0.06149, while the genetic distance between other populations was greater than 0.1 (Table 4). For the *D-loop* gene, the final alignment length was 383 bp. The results of the neutral test indicated that only the *P. argenteus* and *P. echinogaster* populations exhibited negative values, while no significant differences were found among the six populations. The results of the mismatch analysis revealed that only the *P. chinensis* population displayed a unimodal distribution of pairwise differences, while the others did not. Regarding interspecies analysis of the *D-loop* gene, the genetic distance between *P. argenteus* and *P. echinogaster* was the lowest (0.00045), while that between *P. chinensis* and *P. minor* was the largest (0.62412). The genetic distance between *P. cinereus* and *P. punctatissimus* or *P. chinensis* and *P. chinensis* and *P. punctatissimus* ranged from 0.04476 to 0.06596. The genetic distance between *P. minor* and *P. echinogaster* or *P. argenteus* ranged from 0.11211 to 0.11193, while the genetic distance between other populations was greater than 0.6 (Table 3 and Table 4, and Figure S2). For nuclear *S7* gene, the 429 bp gene sequences of six *Pampus* populations were successfully amplified. The results of the neutral test showed that only the Tajima’s D test value of *P. argenteus* population was negative, the Fu’s Fs values of the six *Pampus* populations were positive, and *P. cinereus* and *P. echinogaster* populations exhibited significant differences (Table 3). Mismatch analysis results revealed that only the *P. argenteus* and *P. chinensis* populations were unimodal distributions of pairwise differences (Figure S3). Additionally, the genetic distances between *P. argenteus* and *P. echinogaster* populations were still the lowest among these six *Pampus* populations (0.01889), while the genetic distances between *P. chinensis*, *P. cinereus*, and *P. punctatissimus* populations were relatively close, ranging from 0.02 to 0.03 (Table 4). Overall, based on the three molecular markers, the pairwise genetic distances between *P. argenteus*, *P. minor*, and *P. echinogaster* populations were relatively small, and the same was true for the remaining three *Pampus* species.

The BI and ML analyses yielded almost identical tree topologies based on the *COI* gene among the six *Pampus* populations, indicating high levels of Bayesian posterior probability (PP) values and ML bootstrap values. The trees showed that *Pampus* was divided into two main clades, with *P. argenteus* and *P. echinogaster* being sister groups and forming one clade with *P. minor*. In the other clade, *P. cinereus*, *P. punctatissimus*, and *P. chinensis* were grouped together, with the phylogenetic relationship between *P. cinereus* and *P. punctatissimus* being closer than that between *P. cinereus* and *P. chinensis* (Figures S4 and S5). Similarly, BI and ML analyses based on the *D-loop* gene produced similar results, with high PP values and ML bootstrap values. The results of two phylogenetic trees showed that the six *Pampus* populations were also divided into two main clades. *P. argenteus* and *P. echinogaster*, together with *P. minor*, formed one clade, with the phylogenetic relationship between *P. argenteus* and *P. echinogaster* being closer than that between *P. argenteus* and *P. minor*. *P. cinereus* and *P. punctatissimus*, together with *P. chinensis*, formed the other clade, with the phylogenetic relationship between *P. cinereus* and *P. punctatissimus* being closer than that between *P. cinereus* and *P. chinensis* (Figures S6 and S7). Based on nuclear *S7* gene, the BI and ML analyses generated almost identical tree topologies, and the clustering results were basically consistent with the analysis results of *COI* and *D-loop* molecular markers (Figures S8 and S9). Of note, the BI and ML trees constructed based on the nuclear S7 gene showed strong support for the monophyly in *P. punctatissimus*, *P. cinereus*, *P. chinensis*, and *P. minor* populations, and the samples of *P. argenteus* and *P. echinogaster* populations were still randomly clustered together. Additionally, the two MP trees based on *COI* and *D-loop* genes obtained the same results, and the parameters of the MP tree based on the *COI* sequence were as follows: TL was 418, CI was 0.694 (0.653), RI was 0.988 (0.988), and RC was 0.685 (0.645) for all sites and parsimony-informative sites (in parentheses), while the parameters of the MP tree based on the *D-loop* were as follows: TL was 466, CI was 0.805 (0.757), RI was 0.994 (0.994), and RC was 0.800 (0.752). The topological structures of these two MP trees suggested that *P. argenteus* and *P. echinogaster* were grouped together, and the remaining four *Pampus* species were clustered into another clade. Among them, *P. chinensis* and *P. punctatissimus* were first gathered into one clade, followed by *P. cinereus* and finally *P. minor* (Figures S10 and S11). For the MP tree based on nuclear *S7* gene, *P. cinereus*, *P. chinensis* and *P. punctatissimus* were first clustered together, followed by a mixed cluster of *P. argenteus* and *P. echinogaster*, and finally *P. minor* (Figure S12). The parameters of the MP tree were as follows: TL was 479, CI was 0.624 (0.514), RI was 0.949 (0.949), and RC was 0.593 (0.487).

### 3.3. Comparative Analysis of Mitochondrial Genomes

#### 3.3.1. Mitogenome Organization and Composition

The mitogenomes of *P. argenteus*, *P. punctatissimus*, *P. cinereus*, *P. chinensis*, *P. echinogaster*, *P. minor*, and *P. liuorum* were completely and accurately sequenced. The length ranged from 16,487 bp (*P. liuorum*) to 17,705 bp (*P. minor*), and the A + T content ranged from 56.59% (*P. chinensis*) to 61.01% (*P. minor*) (Table S1). For relatively conserved tRNAs, we found that noncanonical match base pairs or mismatch base pairs were common in tRNAs of the seven *Pampus* species, and internal loops in rRNA usually contained a high proportion of adenosines. The numerous G-U mispairs could hint at their relevance for the specific interaction of the respective RNA with corresponding proteins and/or further RNAs [38]. The mitochondrial tRNAs of all *Pampus* species except for the deletion of the DHU arm in *trnS1* of *P. minor* could be folded into the stable clover-leaf secondary structure (Figure S13). The *Pampus* mitogenomes contained 13 PCGs, of which only the *ND6* gene was encoded on the L-strand, while the remaining 12 PCGs were encoded on the H-strand. Subsequently, we observed that the *COI* gene in these *Pampus* fishes begins with the start codon GTG, a unique feature also observed in other teleost fishes [39,40]. However, there were some differences among the various *Pampus* species. For instance, the start codons of the *ATP6* and *ND4* genes of *P. punctatissimus*, *P. cinereus*, and *P. chinensis* were CTG and GTG, CTG and GTG, and ATA and GTG, respectively, while the start codon of *ND1* in *P. minor* was ATT and GTG for *ND6*. Furthermore, the stop codons of the 13 PCGs in these *Pampus* species were TAA, TAG, and T. It is worth noting that, in addition to the normal stop codons, we also observed incomplete stop codons in some cases, which may be completed after posttranscriptional polyadenylation, a common phenomenon observed in metazoan mitogenomes [41].

#### 3.3.2. Codon Usage Bias and Cluster Analysis

**Figure 3:**
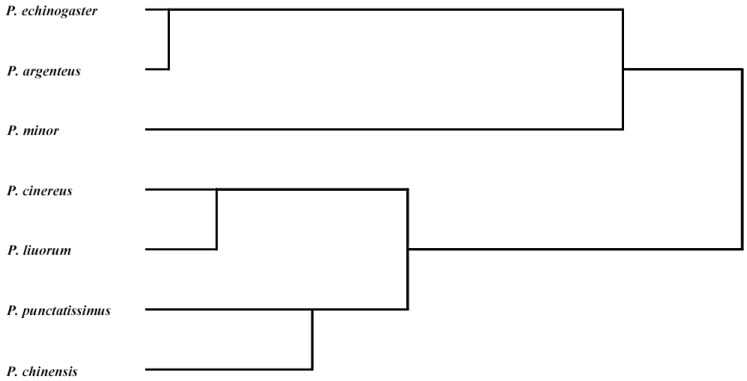
Schematic illustration of the cluster analysis based on the RSCU values of codons.

The number of codons with RSCU values greater than 1 in *P. argenteus*, *P. punctatissimus*, *P. cinereus*, *P. chinensis*, *P. echinogaster*, *P. minor*, and *P. liuorum* was 30, 31, 30, 28, 29, 29, and 31, respectively (Table S2). Moreover, the optimal codons of *Pampus* fishes were determined, as shown in Table S3, where asterisked codons indicate the optimal codons. Based on RSCU values, a cluster analysis was performed, and the results indicated that *P. argenteus* was closely related to *P. echinogaster* with a high bootstrap value. *P. cinereus* and *P. liuorum* were grouped into one clade, and the phylogenetic relationship between the two *Pampus* fishes was closer than that between *P. chinensis* and *P. punctatissimus* (Figure 3).

#### 3.3.3. Mitochondrial Gene Rearrangement

**Figure 4:**
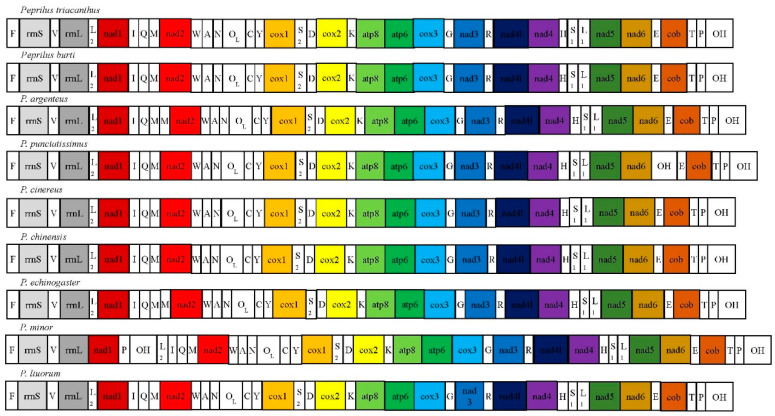
Gene arrangement of mitochondrial genomes from the genus *Pampus*.

The gene rearrangement information of the existing Stromateidae mitogenomes was compared, and the results showed that the gene rearrangements were relatively conservative and involved only tRNAs and OH regions. When the structure of the mitogenomes of other teleost fishes, such as *Peprilus burti* and *Peprilus triacanthus*, was compared, it was found that some mitogenomes of the *Pampus* species had undergone gene recombination. The gene rearrangement regions of the seven *Pampus* species were mainly concentrated near the WANCY gene cluster, IQM gene cluster, and *nad6* gene. An additional and adjacent *trnM* repeat was found in the downstream region of the IQM gene cluster of *P. argenteus* and *P. echinogaster*, and an additional and adjacent OH repeat was found in the downstream region of the *nad6* gene of *P. punctatissimus*. Moreover, the gene rearrangement of *P. cinereus*, *P. chinensis*, and *P. liuorum* was consistent with the mitogenome of classical teleosts, while *P. minor* had a different gene rearrangement. Specifically, the gene rearrangement of *P. minor* was rrnL-nad1-P-OH-L2-I-Q-M, whereas the other gene rearrangements remained unchanged (Figure 4).

#### 3.3.4. Phylogenetic Analysis and Divergence Time Estimation

**Figure 5:**
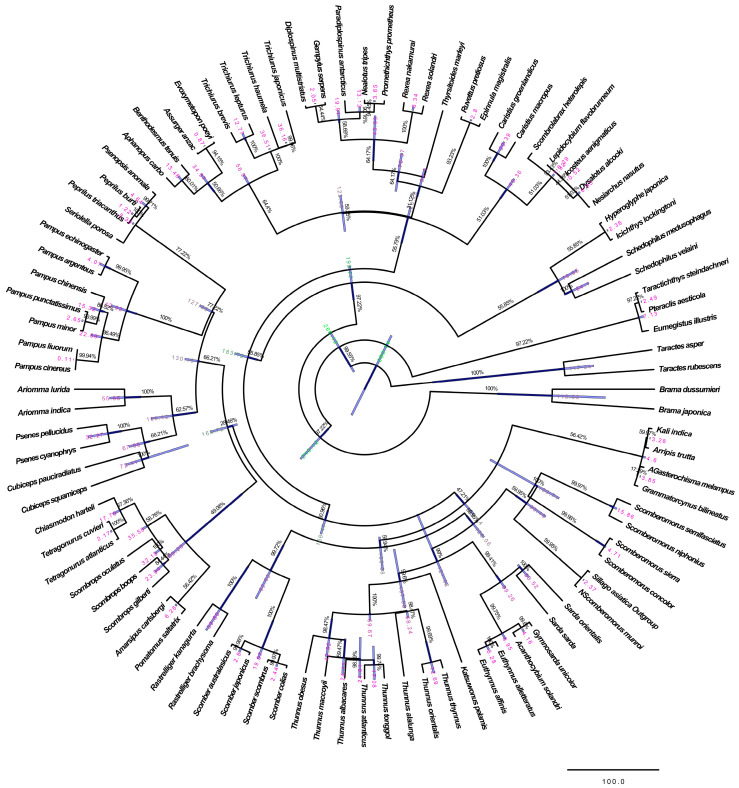
Divergence time estimates for *Pampus* species based on the complete mitochondrial genomes. Blue bars indicate 95% highest posterior density intervals (HPD) for nodes of interest. Numbers in the nodes correspond to age estimates for the major clades. Numbers at nodes correspond to posterior probabilities for nodes.

The BI and ML analyses produced identical branching orders with high PP and bootstrap support values. For MP analyses, TL was 126,546, CI was 0.224 (0.180), RI was 0.461 (0.461), and RC was 0.103 (0.083) for all sites and parsimony-informative sites (in parentheses). All three tree topologies revealed that the clustering consistency of the seven *Pampus* species was relatively high. *P. argenteus* and *P. echinogaster* first formed one cluster and showed the closest relationship to each other, along with *P. minor*, and then they formed the other cluster. The phylogenetic relationship between *P. cinereus* and *P. liuorum* was relatively close, as was the relationship between *P. chinensis* and *P. punctatissimus*, and these four species formed a major cluster (Figures S14–S16). The Bayesian inference analysis using a relaxed molecular clock yielded the same topology as the ML and BI analyses, with strong support (Figure 5). The dated topology indicated that the genus *Pampus* diverged approximately 42.93 million years ago (Mya) with 95% highest posterior density intervals (HPD) of 28.04–57.13 Mya, during the Palaeocene. The divergence time of *P. chinensis* was estimated to be approximately 15.32 Mya (95% HPD: 8.60–23.12), while both *P. argenteus* and *P. echinogaster* diverged approximately 4.01 Mya (95% HPD: 0.10–12.45), occurring mainly in the Pliocene epoch. Additionally, both *P. minor* and *P. punctatissimus* diverged approximately 2.65 Mya (95% HPD: 1.78–3.39), and both *P. cinereus* and *P. liuorum* diverged approximately 0.11 Mya (95% HPD: 0.10–0.13).

### 3.4. Species Delimitation

Using the ABGD method, the species delimitation results based on the *COI* gene showed that the six *Pampus* populations were divided into three groups, among which 36 *P. cinereus* were in group 1, 40 *P. argenteus* and 35 *P. echinogaster* were in group 2, and 36 *P. punctatissimus*, 36 *P. cinereus*, and 34 *P. chinensis* were in group 3. In addition, the other results based on the *D-loop* gene showed that the six *Pampus* populations were divided into eight groups, of which 28 *P. cinereus* were in group 1, eight *P. cinereus* were in group 2, eight *P. punctatissimus* were in group 3, 28 *P. punctatissimus* were in group 4, 40 *P. argenteus* and 35 *P. echinogaster* were in group 5, 32 *P. minor* were in group 6, four *P. minor* were in group 7, and 34 *P. chinensis* were in group 8. Nuclear *S7* gene showed that six *Pampus* populations were divided into 11 groups, 36 *P. punctatissimus* were in group 1, 36 *P. minor* were in group 2, 34 *P. chinensis* were in group 3, 36 *P. minor* were divided into three groups, and the remaining 40 *P. argenteus* and 35 *P. echinogaster* were randomly divided into five groups. Based on the complete mitogenomes of the seven *Pampus* species, the results indicated that *P. argenteus* and *P. echinogaster* were in the same group, while the mitogenome sequences of the other five *Pampus* were in five separate groups. The ASAP method was also used for species delimitation, and the analysis results based on the *COI* gene showed that the six *Pampus* populations were divided into seven subsets, of which 36 *P. cinereus* were in subsets 1, 40 *P. argenteus* and 35 *P. echinogaster* were in subsets 2, 36 *P. punctatissimus* were in subsets 3, 36 *P. minor* were in subset 4, 29 *P. chinensis* were in subset 5, 2 *P. chinensis* were in subset 6, and 3 *P. chinensis* were in subset 7. The results based on the *D-loop* gene showed that the six *Pampus* populations were divided into six subsets, of which 36 *P. cinereus* and 8 *P. punctatissimus* were in subset 1, 28 *P. punctatissimus* were in subset 2, 40 *P. argenteus*, 35 *P. echinogaster* were in subset 3, 32 *P. minor* were in subset 4, 4 *P. minor* were in subset 5, and 34 *P. chinensis* were in subset 6. Based on the analysis results of the nuclear *S7* gene, we found that the ASAP method yielded the same results for species identification as the ABGD method. Based on the mitogenomes, *P. argenteus* and *P. echinogaster* were placed in the same subset, while the mitogenome sequences of the other five *Pampus* were divided into five separate subsets. Additionally, the analysis results of the PTP method all converged. According to the results of the maximum likelihood partition and most supported partition found by a simple heuristic search, the six *Pampus* populations were classified into three categories based on the *COI* gene. Specifically, 36 *P. cinereus*, 36 *P. punctatissimus* and 34 *P. chinensis* were grouped in category 1 (support = 0.791), 40 *P. argenteus* and 35 *P. echinogaster* were grouped in category 2 (support = 0.639), and 36 *P. minor* were grouped in category 3 (support = 0.639). Based on the *D-loop* gene, the results of the maximum likelihood partition showed that the six *Pampus* populations were divided into 95 species, of which 36 *P. minor* were classified into a complete population, and the other *Pampus* populations were disordered. According to the analysis results of nuclear *S7* gene, the six *Pampus* populations were divided into 148 species. The analysis results based on the mitogenomes showed that the six *Pampus* populations were divided into four categories, with *P. argenteus* and *P. echinogaster*, *P. liuorum*, *P. cinereus*, *P. chinensis*, and *P. punctatissimus* were classified into the same species, and *P. minor* was classified as a single species.

## 4. Discussion

### 4.1. Phenotypic Discrimination of Bone Specimens

Fish skeletal information is considered a reliable tool for studying the phylogeny of fish because it remains relatively stable and is not easily influenced by external factors such as growth time and environment. Numerous studies have utilized this basic element to explore the skeletal characteristics of different fish species. For example, Zhang et al. (2000) compared the skeletal characteristics of two *Trachinotus* species and concluded that *T. ovatus* and *T. blochii* were different and distinct species [42]. Yang et al. (2014) analyzed the skeletal system of four fish species, namely, *Ariomma indica*, *Pampus. Argenteus*, *Peprilus triacanthus*, and *Psenopsis anomala*. They discovered that the skull, pharynx, and appendage bones of these species exhibited significant variations, which could be used to distinguish different species [43]. Chen et al. (2014) also utilized the comparison method of bone morphology to compare the skeletal systems of eight sparid species found offshore in China. They found that *Pagrus major* and *P. auratus* belonged to the same species, as did *Acanthopagrus schlegelii schlegelii* and *A. schlegelii czerskii* [44]. The comparison method of bone morphology is a popular approach among fish taxonomists. In this study, we compared 29 bone structures, including the mandibular arch, the hyoid arch, the pectoral fin, the opercular series, and the periorbital bone. Based on these findings, we developed a detailed identification key for differentiating between the *Pampus* species, which is presented in Table 5. However, it was not possible to distinguish between *P. argenteus* and *P. echinogaster* based on the skeletal characteristics.

### 4.2. Population Genetics

Genetic distance is a measure used to quantify the degree of genetic variation between species or populations within the same species and is measured by some numerical value. Before conducting population genetic analysis, the sequences of the experimental samples were verified to belong to a single population based on the nuclear *S7* gene. Here, based on analyses of the *COI*, *D-loop*, and nuclear *S7* genes, we found that the genetic distances between *P. argenteus* and *P. echinogaster* populations was 0.00251, 0.00045, and 0.01889, respectively, which were all below the conventional threshold of 2%. The remaining *Pampus* populations showed genetic distances much greater than 2%. However, it was meaningless to define species only by the parameters of genetic distance, ignoring the evolutionary relationship between species. To accurately define the phylogenetic relationships between the six *Pampus* species, we employed the species delimitation method based on the topological structures of the phylogenetic trees, and the results indicated that the topological structures of these BI and ML phylogenetic trees were similar, with two main branches. Among them, *P. argenteus* and *P. echinogaster* populations were clustered together in one branch, along with *P. minor*. This finding was consistent with previous studies conducted by Divya et al. (2017) and Yin et al. (2019) [4,6]. In the other main branch, *P. cinereus* and *P. punctatissimus* were initially clustered together, followed by clustering with *P. chinensis* to form the other main branch. These results were consistent with the findings of Wei et al. (2021) [45]. Subsequently, the MP trees constructed based on the *COI* and *D-loop* genes obtained the same result, but it was different from the BI and ML trees constructed based on the same molecular data. The clustering results of *P. cinereus*, *P. chinensis*, and *P. punctatissimus* showed similar differences in BI and ML analysis compared to the analysis results of the MP method. There were a few samples of *P. cinereus* or *P. chinensis* in the clusters mixed into *P. punctatissimus*, and the difference in genetic distance between *P. cinereus* and *P. punctatissimus*, *P. chinensis*, and *P. punctatissimus* was only 0.002. This may be due to the relatively similar genetic relationships among these three *Pampus* species. However, the cluster branches of *P. minor* changed greatly, which may be due to the high degree of sequence differentiation between the *P. minor* and other fishes of the genus *Pampus*, as proven by the value of genetic distance between them. Differently, the topological structures of the MP, BI, and ML trees based on the nuclear *S7* gene had the same result. This indicated that the nuclear *S7* gene may have a more sensitive identification ability for species identification of the six *Pampus* species.

Additionally, we applied the *COI*, *D-loop*, and nuclear *S7* gene molecular markers to classify the *Pampus* species and evaluated taxonomic units using ABGD and ASAP, PTP methods. These results all showed that both *P. argenteus* and *P. echinogaster* were considered the same species, and *P. minor* was a valid species. The difference was that the species delimitation of *P. cinereus*, *P. punctatissimus*, and *P. chinensis* was chaotic based on *COI* and *D-loop* molecular markers, while the nuclear *S7* gene molecular marker could clearly identify these species. From this, it can be seen that *COI* and *D-loop* molecular markers were not suitable for the differentiation of *P. cinereus*, *P. punctatissimus*, and *P. chinensis*, except for *P. argenteus*, *P. echinogaster*, and *P. minor.* The limited genetic information content may not have been suitable for species delimitation of *Pampus* to some extent. As a result, it was necessary to classify them more reliably through more genetic information.

### 4.3. Phylogenetic Interrelationships

Codons are essential components of protein translation in gene-coding regions. Analyzing the characteristics of codon usage in gene-coding regions is of great significance for studying gene function and phylogenetics [46]. In this study, the phylogenetic results based on mitogenomes were consistent with the cluster analysis results based on RSCU values. This result indicated that the CUB in the *Pampus* mitogenomes may be related to their genetic relationship, and may also trace a different evolutionary path from species evolution [47]. *P. argenteus* and *P. echinogaster* may have the same evolutionary history and closer genetic relationship than the other *Pampus* fishes. Furthermore, some studies have shown that sequence evolution may also be related to gene arrangement [48]. Generally, fish mitogenomes are known for their highly conserved organization, especially in the order of gene arrangement. However, there have been no systematic studies on gene rearrangement in *Pampus* mitogenomes. In this study, an additional and adjacent *trnM* repeat was found in *P. argenteus* and *P. echinogaster*, and the two *Pampus* fishes have an identical order of gene arrangement. The *trnL2* gene of *P. minor* was translocated. Additionally, there was also a gene duplication of *trnP* and a 379 bp noncoding region. For the remaining *Pampus* species, except for an additional and adjacent OH repeat found in *P. punctatissimus*, the others were consistent with the evolution of mitochondrial gene rearrangements among teleost species. These gene rearrangements mainly occurred in the WANCY gene cluster, IQM gene cluster, *nad6*, *D-loop* and their adjacent genes, and these regions have often been studied in mitogenomes of other fishes. For example, in the *Cynoglossus* subfamily represented by *C. semilaevis*, most of the control regions were translocated to the 3′end of *ND1*, and the position of the *Q* gene was changed from the light to the heavy strand. These changes were accompanied by shuffling of the *I* gene and long-range translocation of the putative control region downstream to a site between the *ND1* and *Q* genes [49]. The control region of *Lampetra fluviatilis* and *Petromyzon marinus* was located between the *ND6* and *Cytb* genes, rather than between the *Cytb* and *12S* genes [50,51]. Gene rearrangements were observed in various deep-sea benthic fishes such as *Monognathus jesperseni*, *Saccopharynx lavenbergi*, and *Eurypharynx pelecanoides* [52,53]. It may be that during the evolutionary history of *Pampus* mitogenomes, various selection pressures caused constant mutations in the genome sequence, resulting in changes in genome structure. These natural selection effects could have caused multiple gene rearrangements at the genome level [53].

Additionally, the divergence time of the genus *Pampus* started in the Palaeocene. Teleostei experienced significant expansion in the late Cretaceous period and established their dominant species in rivers, lakes, and ocean. In the Pliocene, fish underwent many changes, with the most important being the evolution of teleosts, which appeared in many important branches. In the Pleistocene epochs, some fish species adapted to environmental changes and human activities, which may be closely related to the fact that the climate environment was conducive to the species formation and radiation evolution of *Pampus*. The divergence time of the *Pampus* species was found to be similar to that of most teleost species. Moreover, the three species delimitation methods were conducted to distinguish the genus *Pampus* species using complete mitogenomes. Similarly, both *P. argenteus* and *P. echinogaster* were also considered the same species, and the difference was that the remaining five *Pampus* species were divided into five categories by using the ABGD and ASAP methods, while they were divided into four categories by using the PTP method. Both *P. argenteus* and *P. echinogaster*, both *P. liuorum* and *P. cinereus*, and both *P. chinensis* and *P. punctatissimus* were classified into the same species, respectively, and *P. minor* was classified as a single species. Zhang et al. (2013) introduced that PTP is a model for delimiting species on a rooted phylogenetic tree, and speciation or branching events are modeled in terms of number of substitutions [35]. So, the results of species definition by PTP method may be caused by the close phylogenetic relationship of these three pairs of *Pampus* species. Combined with bone information as well as nuclear and mitochondrial molecular data, these results all indicated that *P. argenteus* and *P. echinogaster* are the same species, *P. minor* is a valid species, *P. liuorum* is speculated to be a valid species, and *P. cinereus* is closely related to *P. chinensis* and *P. punctatissimus*, but they are different species.

## 5. Conclusions

This study integrated information on the skeletal structure of the genus *Pampus*, population genetic analysis results, and analysis results of the complete mitogenomes. Based on these findings, we suggested that *P. argenteus* and *P. echinogaster* should be classified as the same species, *P. minor* is a valid species, *P. liuorum* should be regarded as an effective species, and *P. cinereus* is closely related to *P. chinensis* and *P. punctatissimus*, but they are different species. In summary, *Pampus* can be divided into six species: *P. argenteus*, *P. punctatissimus*, *P. cinereus*, *P. chinensis*, *P. minor*, and *P. liuorum.* This study provides important basic information for the species delimitation and evolutionary history of *Pampus* species.

## Figures and Tables

**Table 1:** Descriptive statistics of morphological information of the six *Pampus* species.

Index	*P. argenteus*	*P. punctatissmus*	*P. cinereus*	*P. chinensis*	*P. echinogaster*	*P. minor*
Body length/mm	134.15 ± 4.05	154.03 ± 32.42	144.87 ± 6.86	198.13 ± 10.69	130.03 ± 4.39	107.10 ± 10.86
Body weight/g	78.00 ± 9.50	55.33 ± 4.29	159.37 ± 12.66	315.47 ± 30.05	80.07 ± 8.27	59.63 ± 4.94
The number of vertebrae	38–40	34–35	38	32	40	30–31
The number of dorsal ribs	24	15–19	26	19	24	17–18
The number of abdominal ribs	12	12	12	13	14	11
The number of dorsal fin rays	57–60	49–53	42	53–55	59	49

**Table 2:** Skeletal information of the six *Pampus* species.

Skeletal Structure	Bones	*P. argenteus*	*P. punctatissimus*	*P. cinereus*	*P. chinensis*	*P. echinogaster*	*P. minor*
Mandibular arch	Maxilla/premaxilla	The length ratio of the maxilla to the premaxilla is 3:2	The length ratio of the maxilla to the premaxilla is 2:1	The length ratio of the maxilla to the premaxilla is 2:1	The length ratio of the maxilla to the premaxilla is 3:2	The length ratio of the maxilla to the premaxilla is 3:2	The length ratio of the maxilla to the premaxilla is 2:1
	Palatine bone	Short, thick back end	Long, thin back end	Long, thin back end	Short, thick back end	Short, thick back end	Short, thick back end
	Mesopterygoid bone	Short	Long	Long	Long	Short	Short
	Metapterygoid bone	The front end is curved inward in an “L” shape	The front end is curved inward in an “L” shape	The front end is straight	The front end is curved inward in an “L” shape	The front end is curved inward in an “L” shape	The front end is straight
	Quadrate bone	Fan-shaped	Trapezoidal	Trapezoidal	Trapezoidal	Fan-shaped	Fan-shaped
	Dentary bone	90°	60°	60°	90°	90°	90°
	Articular bone	Wide	Narrow	Narrow	Wide	Wide	Wide
	Angular bone	Trapezoidal	Trapezoidal	Trapezoidal	Trapezoidal	Trapezoidal	Trapezoidal
Hyoid arch	Hyomandibular bone	The upper is rounded, and the lower end is thin	The upper is triangular, and the lower is thinner and shorter	The upper is triangular, and the lower is thinner and shorter	The upper is triangular, and the lower is thinner and shorter	The upper is rounded, and the lower end is thin	The upper is triangular, and the lower is thinner and shorter
	Basihyal bone	Irregular and spherical structure	Irregular and spherical structure	Irregular and spherical structure	Irregular and spherical structure	Irregular and spherical structure	Irregular and spherical structure
	Urohyal bone	Sharp	Slightly cupped	Round and blunt	Round and blunt	Sharp	Sharp
	Ceratohyal bone	Long	Long	Long	Long	Long	Long
	Interhyal bone	Small	Big	Big	Big	Small	Small
	Epihyal bone	Triangular	Triangular	Triangular	Triangular	Triangular	Triangular
	Branchiostegal ray	Gradually increased in length from the bottom to the top	Gradually increased in length from the bottom to the top	Gradually increased in length from the bottom to the top	Gradually increased in length from the bottom to the top	Gradually increased in length from the bottom to the top	Gradually increased in length from the bottom to the top
	Hypohyal bone	Large and wide	Large and wide	Large and wide	Large and wide	Large and wide	Large and wide
Opercular series	Preopercular bone	90°	>90°, and the upper end is obviously curved	>90°	>90°, and the upper end is obviously curved	90°	90°
	Opercular bone	90°	60°	60°	60°	90°	90°
	Interopercular bone	Kidney shaped, and curved abdominal margin	Straight abdominal margin	Straight abdominal margin	Straight abdominal margin	Kidney shaped, and curved abdominal margin	Kidney shaped, and curved abdominal margin
	Subopercular bone	Deeply depressed both the upper and lower ends	Deeply depressed both the upper and lower ends	Not depressed inward	Not depressed inward	Deeply depressed both the upper and lower ends	Deeply depressed both the upper and lower ends
Pectoral girdle	Posttemporal	Thin and small both the upper and lower ends	Thin and short upper end and a wide and long lower end	Thin and short upper end and a wide and long lower end	Thin and short upper end and a wide and long lower end	Thin and small both the upper and lower ends	Thin and short upper end and a wide and long lower end
	Supracleithrum	The two ends are blunt	The two ends are longer and narrower	The two ends are longer and narrower	The two ends are longer and narrower	The two ends are blunt	The two ends are blunt
	Cleithrum	The lower end is wide	The lower end is narrow	The lower end is twisted inward	The lower end is narrow	The lower end is wide	The lower end is wide
	Scapula	The scapula hole is narrow	The scapula hole is oval	The scapula hole is oval	The scapula hole is oval	The scapula hole is narrow	The scapula hole is narrow
	Coracoid	Big	Small	Big	Small	Big	Big
	Postcleithrum	Narrow	Wide	Wide	Wide	Narrow	Narrow
	Pelvic girdle	A sharp upper end and a wide lower end	A sharp upper end and a wide lower end	A sharp upper end and a wide lower end	A sharp upper end and a wide lower end	A sharp upper end and a wide lower end	A sharp upper end and a wide lower end
	Periorbital bone	Thin and flat rod-like structure	Thin and flat rod-like structure	Thin and flat rod-like structure	Thin and flat rod-like structure	Thin and flat rod-like structure	Thin and flat rod-like structure

**Table 3:** Genetic variability and neutrality test data from the six *Pampus* species.

Species	Gene	N	Hd (SD)	π	Tajima’s D	Fu’s Fs
*P. argenteus*	*COI*	40	0.796 (0.057)	0.0030	−1.38674	−4.42077 *
*P. punctatissimus*		36	0.625 (0.072)	0.0200	−0.44976	18.92812
*P. cinereus*		36	0.905 (0.022)	0.0041	2.05700	13.85279
*P. chinensis*		34	0.793 (0.056)	0.0269	−0.41256	12.85374
*P. echinogaster*		35	0.908 (0.026)	0.0041	−0.62185	−3.05574
*P. minor*		36	0.157 (0.077)	0.0004	−0.67689	0.88954
*P. argenteus*	*D-loop*	40	0.232 (0.085)	0.0006	−0.92544	−1.15034
*P. punctatissimus*		36	0.848 (0.040)	0.0316	1.26492	9.01636
*P. cinereus*		36	0.908 (0.022)	0.0370	2.76089	5.08509
*P. chinensis*		34	0.692 (0.056)	0.0036	0.34679	0.38654
*P. echinogaster*		35	0.111 (0.070)	0.0003	−0.80662	−0.57234
*P. minor*		36	0.917 (0.018)	0.1045	3.87195	16.95094
*P. argenteus*	*S7*	40	0.944 (0.017)	0.0154	−0.50794	1.19457
*P. punctatissimus*		36	0.916 (0.030)	0.0091	0.71393	1.09758
*P. cinereus*		36	0.635 (0.067)	0.0130	2.01110	1.98272 *
*P. chinensis*		34	0.865 (0.034)	0.0074	0.75482	0.95917
*P. echinogaster*		35	0.899 (0.025)	0.0400	0.91534	1.84045 *
*P. minor*		36	0.632 (0.058)	0.0057	0.67569	1.30771

N: indicates number of specimens; Hd: indicates haplotype diversity; π: indicates nucleotide diversity; SD: indicates standard deviation; *: 0.01 < *p* < 0.05.

**Table 4:** Description statistics of genetic distance between the six *Pampus* populations.

Species	Gene	*P. cinereus*	*P. echinogaster*	*P. punctatissimus*	*P. argenteus*	*P. minor*	*P. chinensis*
*P. cinereus*	*COI*						
*P. echinogaster*		0.11425					
*P. punctatissimus*		0.04726	0.10739				
*P. argenteus*		0.11392	0.00251	0.10682			
*P. minor*		0.10637	0.12596	0.10560	0.12598		
*P. chinensis*		0.06149	0.11408	0.04946	0.11344	0.10779	
*P. cinereus*	*D-loop*						
*P. echinogaster*		0.61399					
*P. punctatissimus*		0.04476	0.60051				
*P. argenteus*		0.61410	0.00045	0.60062			
*P. minor*		0.61635	0.11193	0.60612	0.11211		
*P. chinensis*		0.06596	0.61544	0.06000	0.61555	0.62412	
*P. cinereus*	*S7*						
*P. echinogaster*		0.05738					
*P. punctatissimus*		0.02910	0.06800				
*P. argenteus*		0.06444	0.01889	0.06445			
*P. minor*		0.04688	0.04092	0.04723	0.03302		
*P. chinensis*		0.02768	0.07189	0.02880	0.06082	0.04326	

Note: The lower left corner of the same gene is the value of genetic distance, and the upper right corner is its standard deviation.

**Table 5:** An identification key of the six *Pampus* species.

Key to Species of the Six *Pampus*
1a. The length ratio of the maxilla to the premaxilla is 2:1.
2a. The palatine bone is long, and the rear ends of that is thin. The quadrate bone is trapezoidal. The angle of the dentary bone is about 60°. The angle between the upper and lower ends of the opercular bone is about 60°. The articular bone is narrow. The ventral margin of the interopercular bone is straight. The upper end of the posttemporal is thin and short, and the lower end is wide and large. The hole in the scapula looks like an ellipse.
3a. The front end of the metapterygoid bone bends inward in the shape of an “L”. The lower end of the cleithrum is narrow (*P. punctatissimus*).
3b. The front end of the metapterygoid bone is basically straight. The lower end of the cleithrum is twisted inward (*P. cinereus*).
2b. The palatine bone is short, and the rear ends of that is thick. The quadrate bone is fan shaped. The angle of the dentary bone is about 90°. The angle between the upper and lower ends of the opercular bone is about 90°. The articular bone is wide. The interopercular bone is “kidney” shaped, with the abdominal margin curved. The upper end of the posttemporal is thin and short, and lower end is wide and long. The hole in the scapula is narrow (*P. minor*).
1b. The length ratio of the maxilla to the premaxilla is 3:2.
2c. The quadrate bone is trapezoidal. The upper end of the hyomandibular bone is triangular, and the lower end is thin and short. The angle between the upper and lower ends of the opercular bone is about 60°. The ventral margin of the interopercular bone is straight. The upper end of the posttemporal is thin and small, and the lower end is wide and large. The hole in the scapula looks like an ellipse (*P. chinensis*).
2d. The quadrate bone is fan shaped. The upper end of the hyomandibular bone is round and the lower end is thin. The angle between the upper and lower ends of the opercular bone is about 90°. The interopercular bone is “kidney” shaped, with the abdominal margin curved. The upper and lower ends of the posttemporal are thin and small. The hole in the scapula is narrow (*P. argenteus* or *P. echinogaster*).

## Data Availability

The sequence data have been submitted to GenBank under accession numbers OQ409923–OQ410139, OQ518452–OQ518491, OQ518493–OQ518669, OR538381–OR538387, and OR540873–OR541089.

## Supplementary Materials

The following supporting information can be downloaded at: https://www.mdpi.com/article/10.3390/ani14050814/s1, Figure S1: Mismatch distribution for demographic expansion of the genus *Pampus* based on the *COI* gene; Figure S2: Mismatch distribution for demographic expansion of the genus *Pampus* based on *D-loop* gene; Figure S3: Mismatch distribution for demographic expansion of the genus *Pampus* based on nuclear *S7* gene; Figure S4: Phylogenetic relationships derived from maximum likelihood (ML) based on the *COI* nucleotide sequences of the six *Pampus* populations. Numbers in the nodes are ML bootstrap proportions; Figure S5: Bayesian phylogeny of the genus *Pampus* based on the combined analysis of the *COI* gene marker. The nodes are posterior probability support values; Figure S6: Phylogenetic relationships derived from maximum likelihood (ML) based on the *D-loop* nucleotide sequences of the six *Pampus* populations. Numbers in the nodes are ML bootstrap proportions; Figure S7: Bayesian phylogeny of the genus *Pampus* based on the combined analysis of the *D-loop* gene marker. The nodes are posterior probability support values; Figure S8: Phylogenetic relationships derived from maximum likelihood (ML) based on the nuclear *S7* gene sequences of the six *Pampus* populations. Numbers in the nodes are ML bootstrap proportions; Figure S9: Bayesian phylogeny of the genus *Pampus* based on the combined analysis of the nuclear *S7* gene. The nodes are posterior probability support values; Figure S10: The maximum parsimony tree of the 217 accessions of *Pampus* species based on inferred *COI* sequences. The outgroup was *Sillago asiatica;* Figure S11: The maximum parsimony tree of the 217 accessions of *Pampus* species based on inferred *D-loop* sequences. The outgroup was *Sillago asiatica;* Figure S12: The maximum parsimony tree of the 217 accessions of *Pampus* species based on inferred nuclear *S7* gene sequences. The outgroup was *Thunnus albacares;* Figure S13: Schematic diagram of the secondary structure of all tRNAs in the mitochondrial genome of the genus *Pampus;* Figure S14: The relationships of maximum likelihood of the genus *Pampus* based on the combined analysis of all known mitochondrial genome sequences of Scombriformes from the NCBI database. Numbers in parentheses are SH-aLRT support (%)/aBayes support/ultrafast bootstrap support (%); Figure S15: Bayesian phylogeny of the genus *Pampus* based on the combined analysis of all known mitochondrial genome sequences of Scombriformes from the NCBI database. The nodes are posterior probability support values; Figure S16: The maximum parsimony analysis of the genus *Pampus* based on the combined analysis of all known mitochondrial genome sequences of Scombriformes from the NCBI database; Table S1: Summary of complete mitochondrial gene/element features of *P. argenteus*, *P. punctatissimus*, *P. cinereus*, *P. chinensis*, *P. echinogaster*, *P. minor* and *P. liuorum;* Table S2: Description statistics of the codon number and relative synonymous codon usage (RSCU) in 13 protein coding genes of the genus *Pampus;* Table S3: The optimal codon analysis of the 13 protein-coding genes of the genus *Pampus*.
