# Supplementary material for: Comparative Analysis of the Systematics and Evolution of the Pampus Genus of Fish (Perciformes: Stromateidae) Based on Osteology, Population Genetics and Complete Mitogenomes
Source: Animals (Basel). 2024 Mar 6;14(5):814. doi: 10.3390/ani14050814 (PMC10930999; doi:10.3390/ani14050814)
Supplement: Supplementary file 1 [file animals-14-00814-s001.zip › Supplementary information/Fig. S13.pdf]

Pampus argenteus

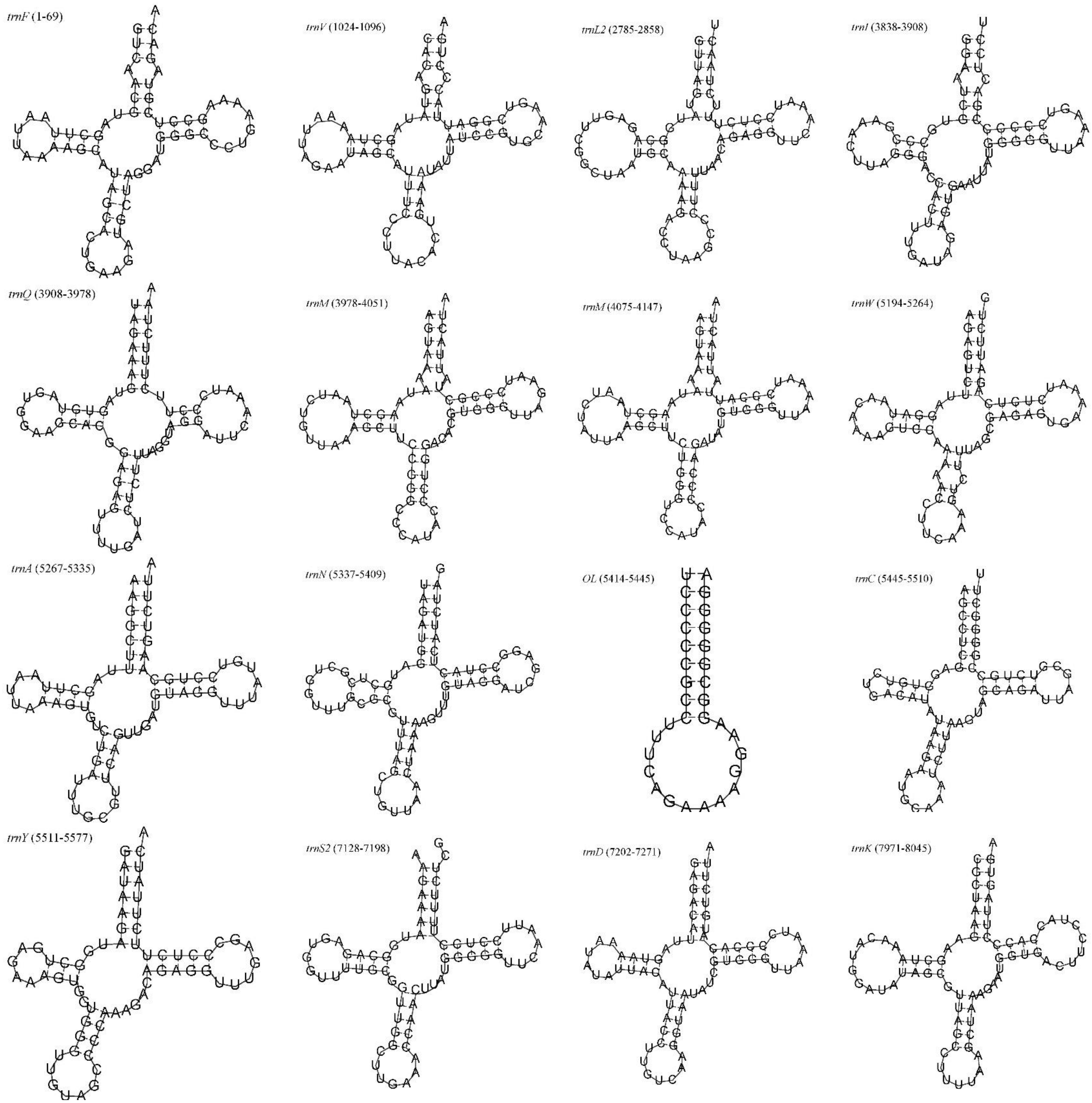

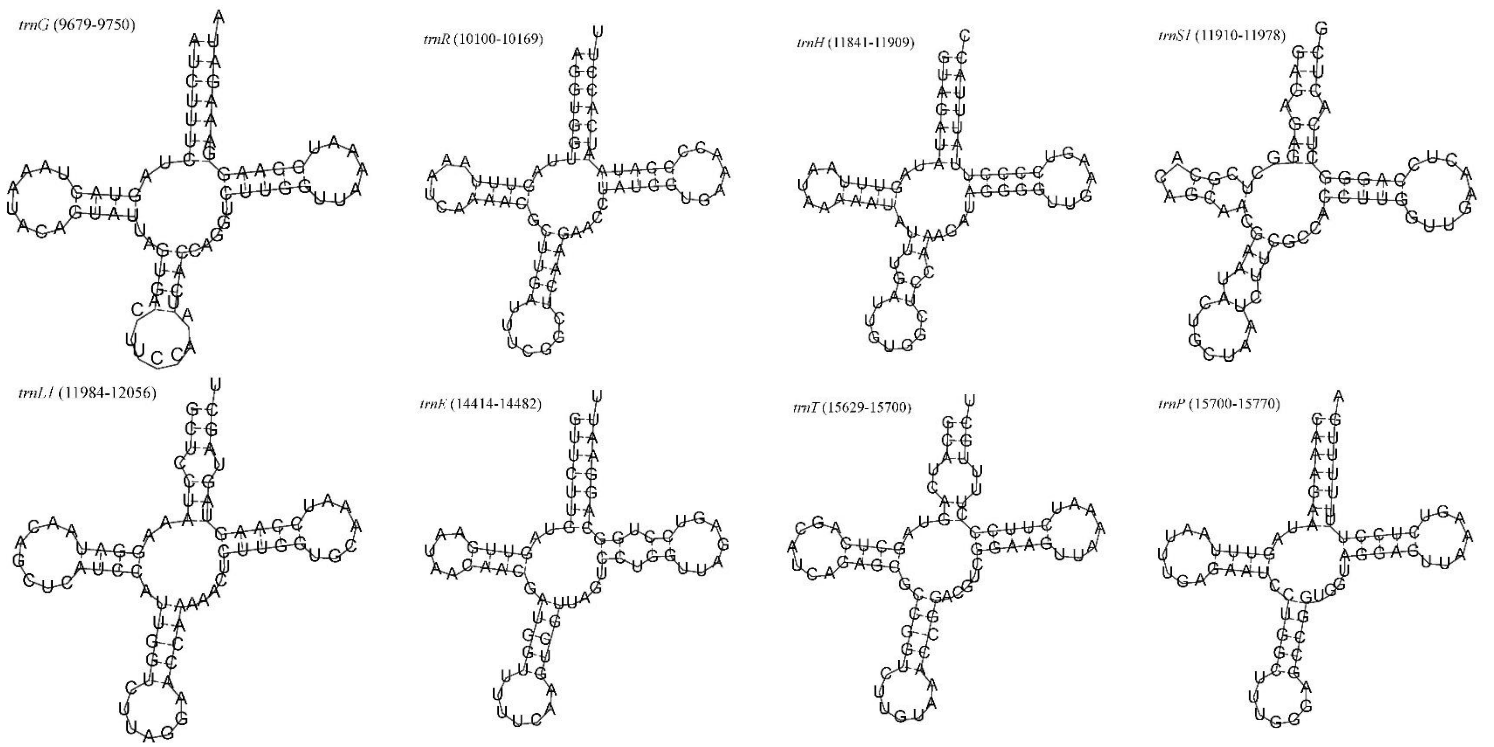
trnG (9679-9750)
trnR (10100-10169)
trnH (11841-11909)
trnS1 (11910-11978)
trnL1 (11984-12056)
trnE (14414-14482)
trnT (15629-15700)
trnP (15700-15770)

Pampus punctatissimus

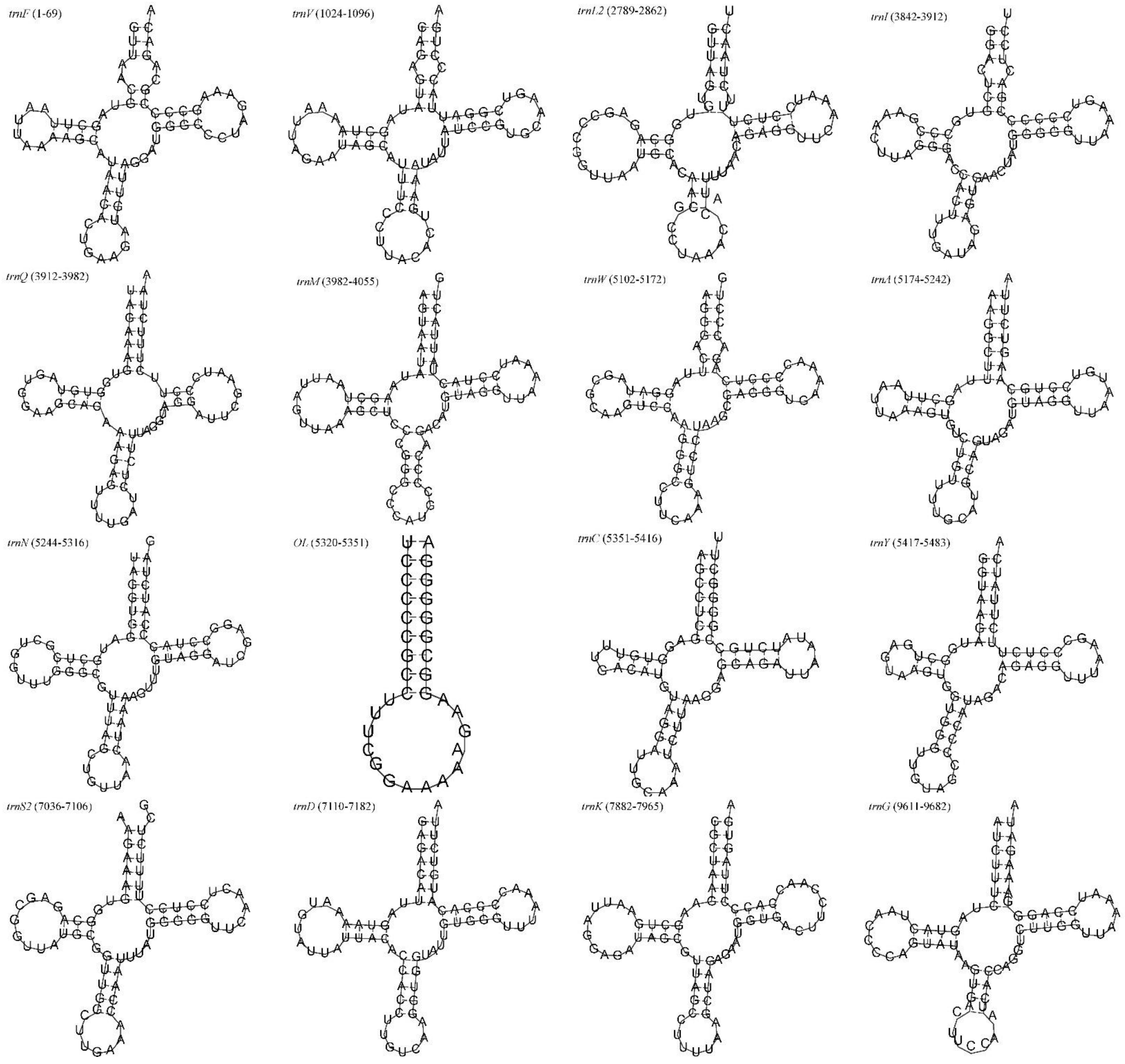

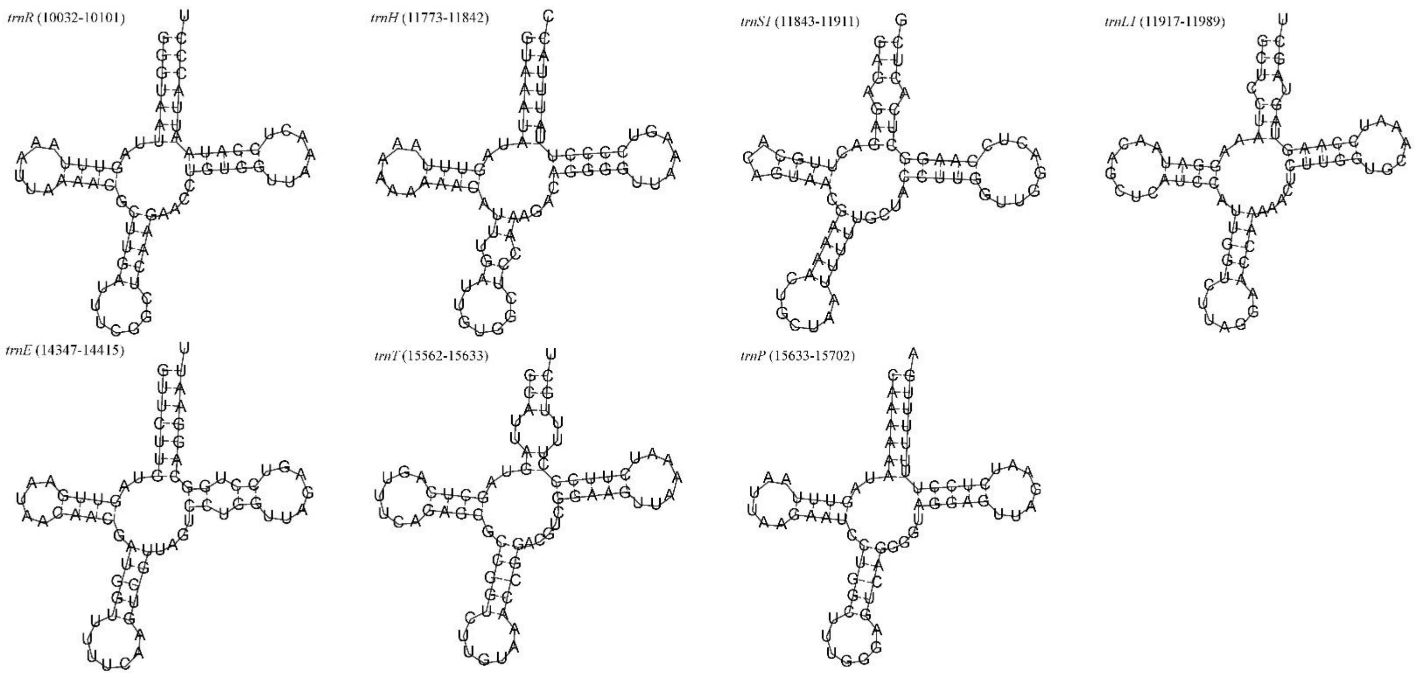
trnR (10032-10101)
trnH (11773-11842)
trnS1 (11843-11911)
trnL1 (11917-11989)
trnE (14347-14415)
trnT (15562-15633)
trnP (15633-15702)

Pampus cinereus

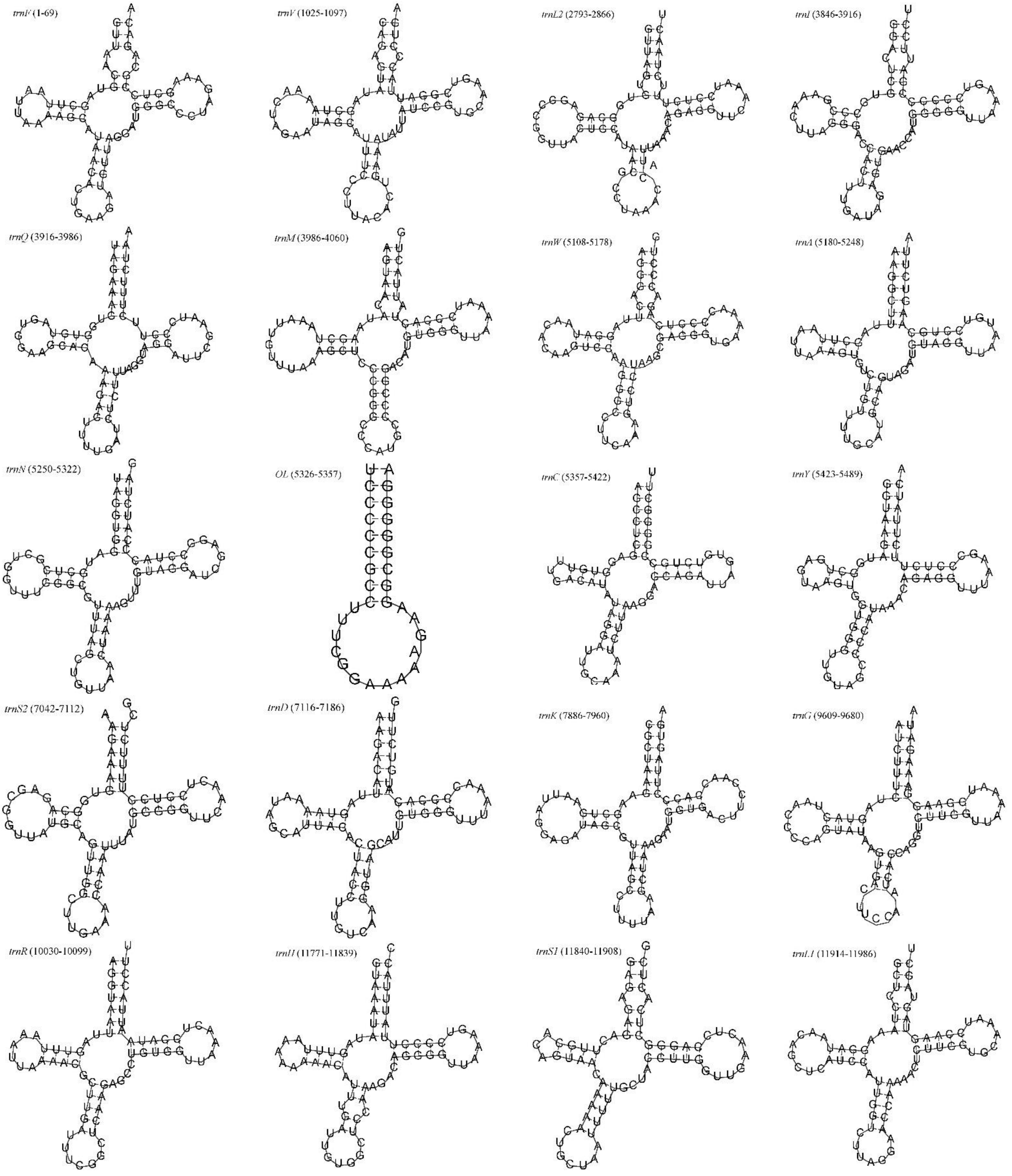

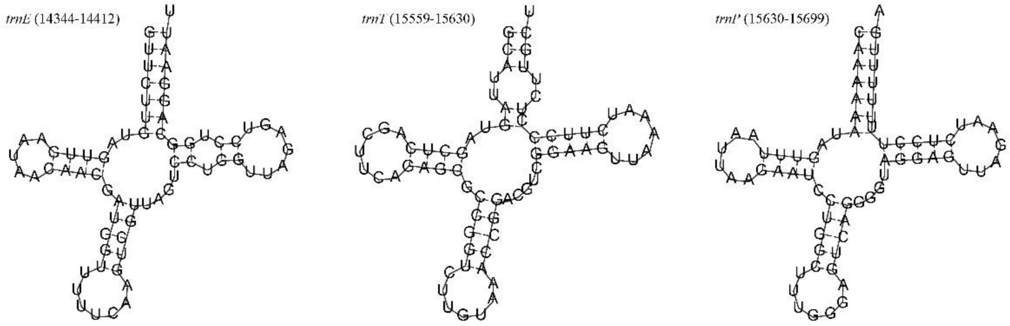

trnE (14344-14412)
trnT (15559-15630)
trnP (15630-15699)

Pampus chinensis

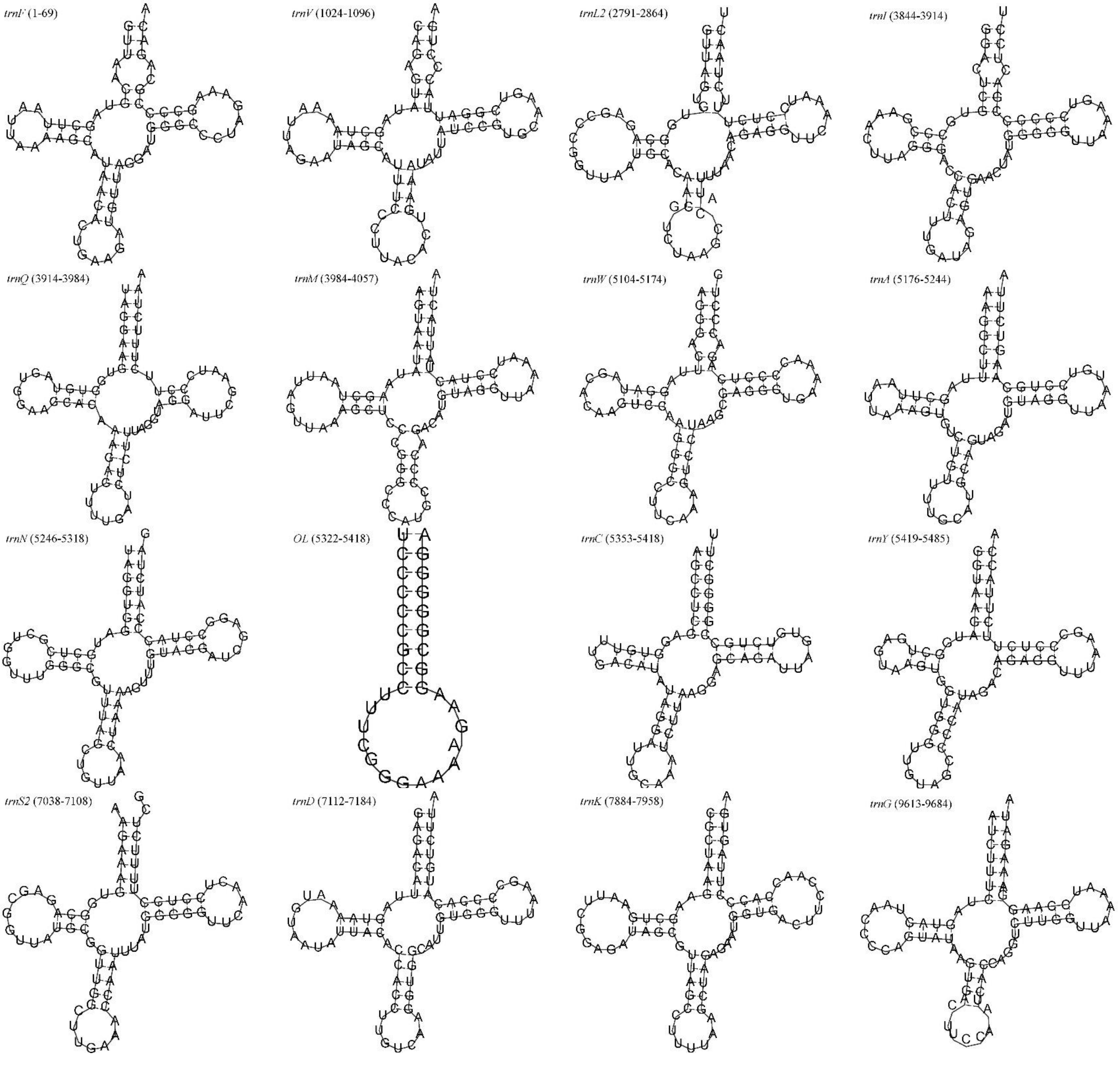

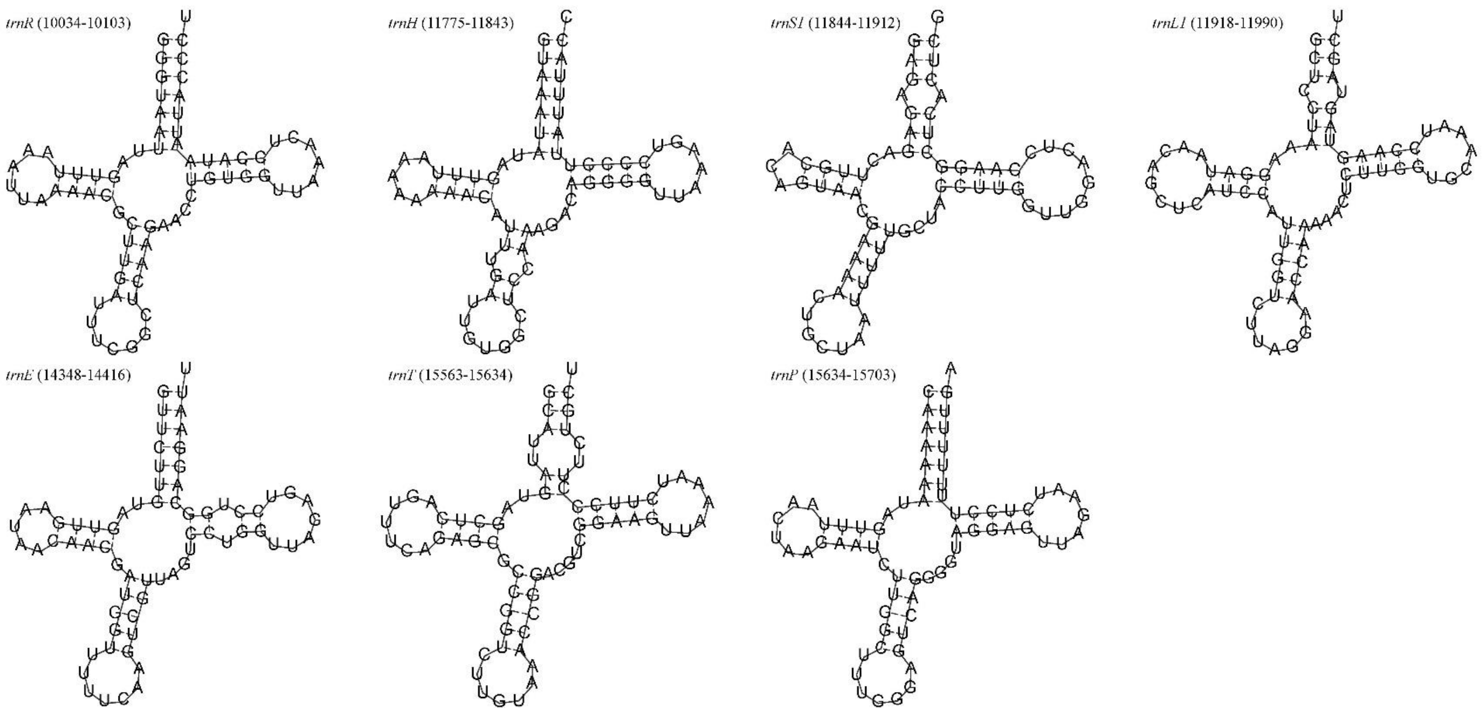
trnR (10034-10103)
trnH (11775-11843)
trnS1 (11844-11912)
trnL1 (11918-11990)
trnE (14348-14416)
trnT (15563-15634)
trnP (15634-15703)

Pampus echinogaster

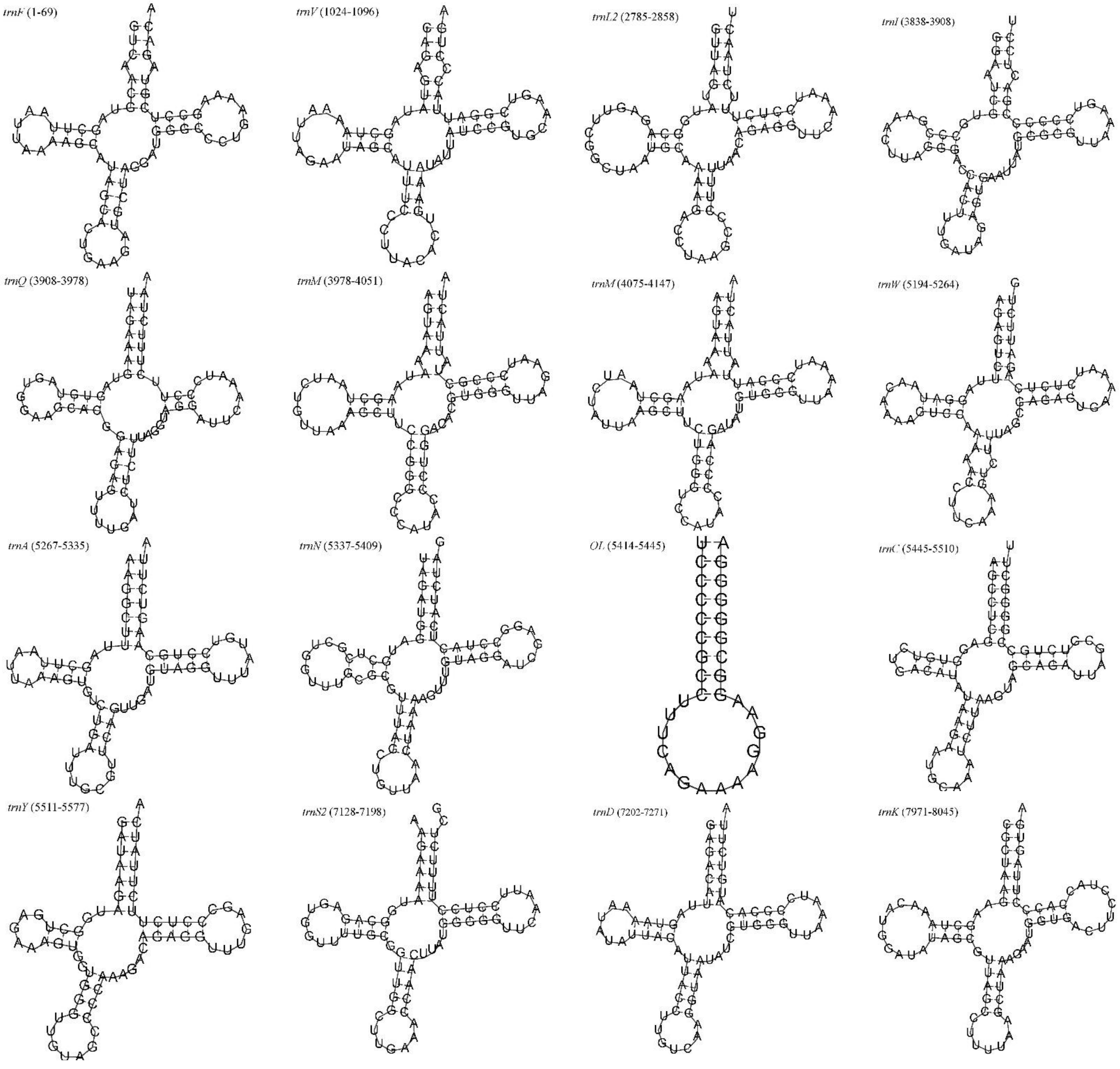

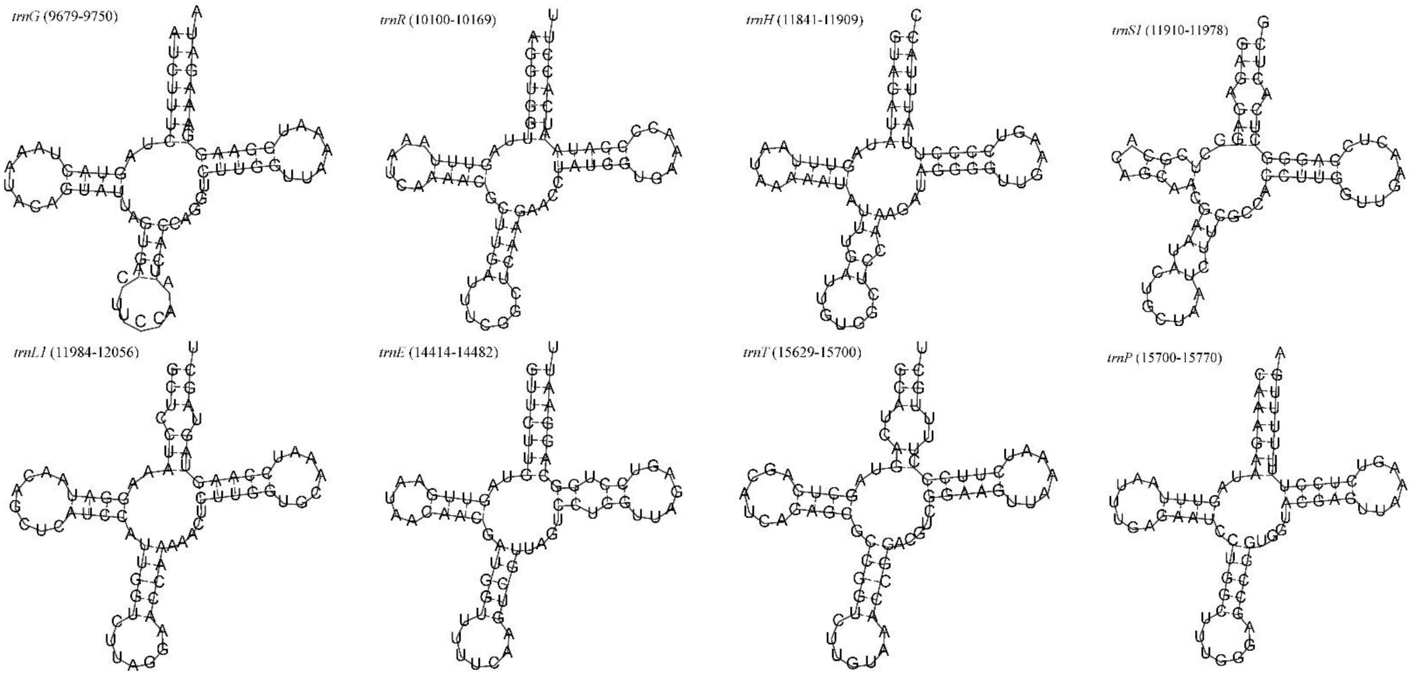
trnG (9679-9750)
trnR (10100-10169)
trnH (11841-11909)
trnS1 (11910-11978)
trnL1 (11984-12056)
trnE (14414-14482)
trnT (15629-15700)
trnP (15700-15770)

Pampus minor

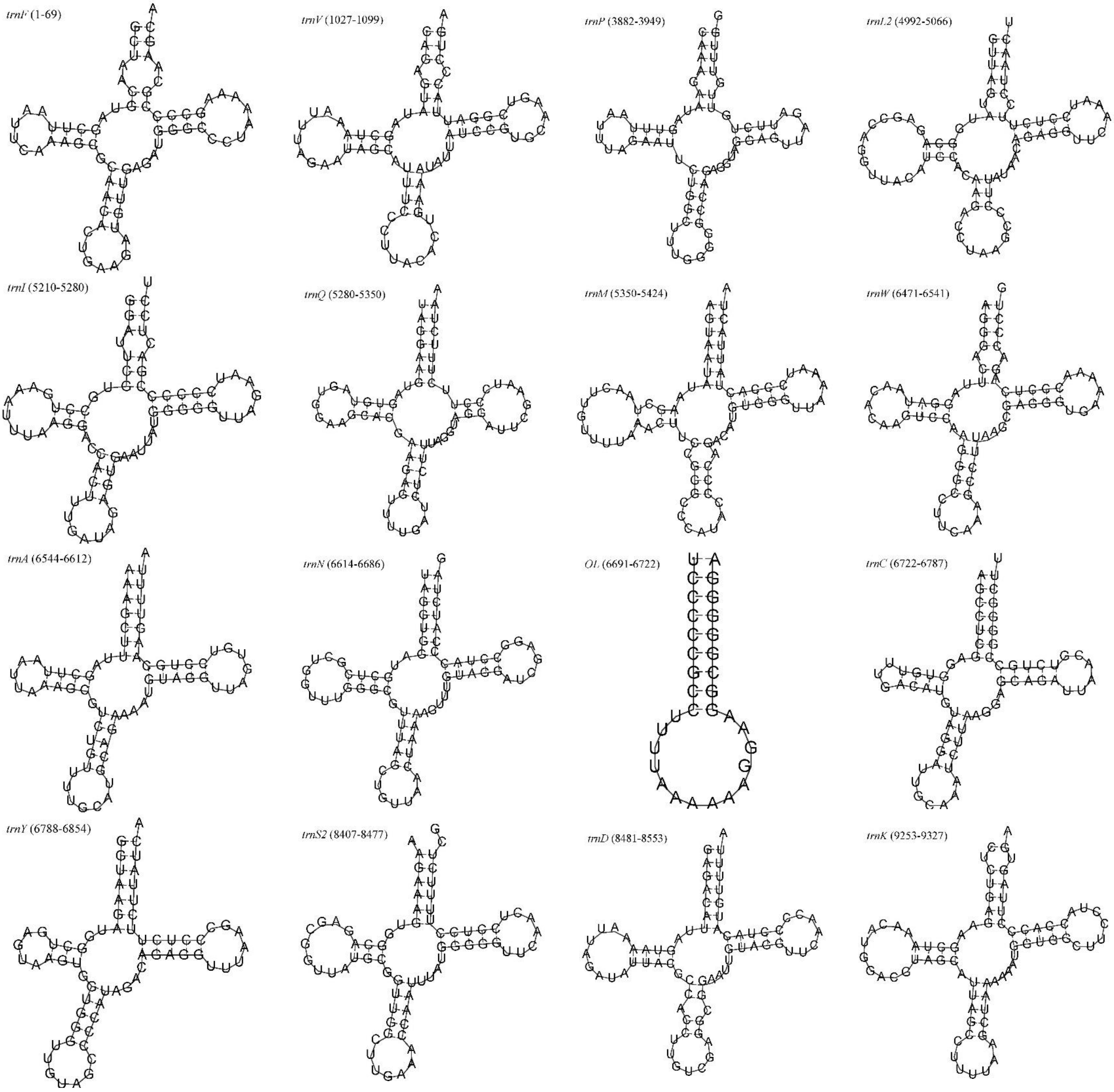

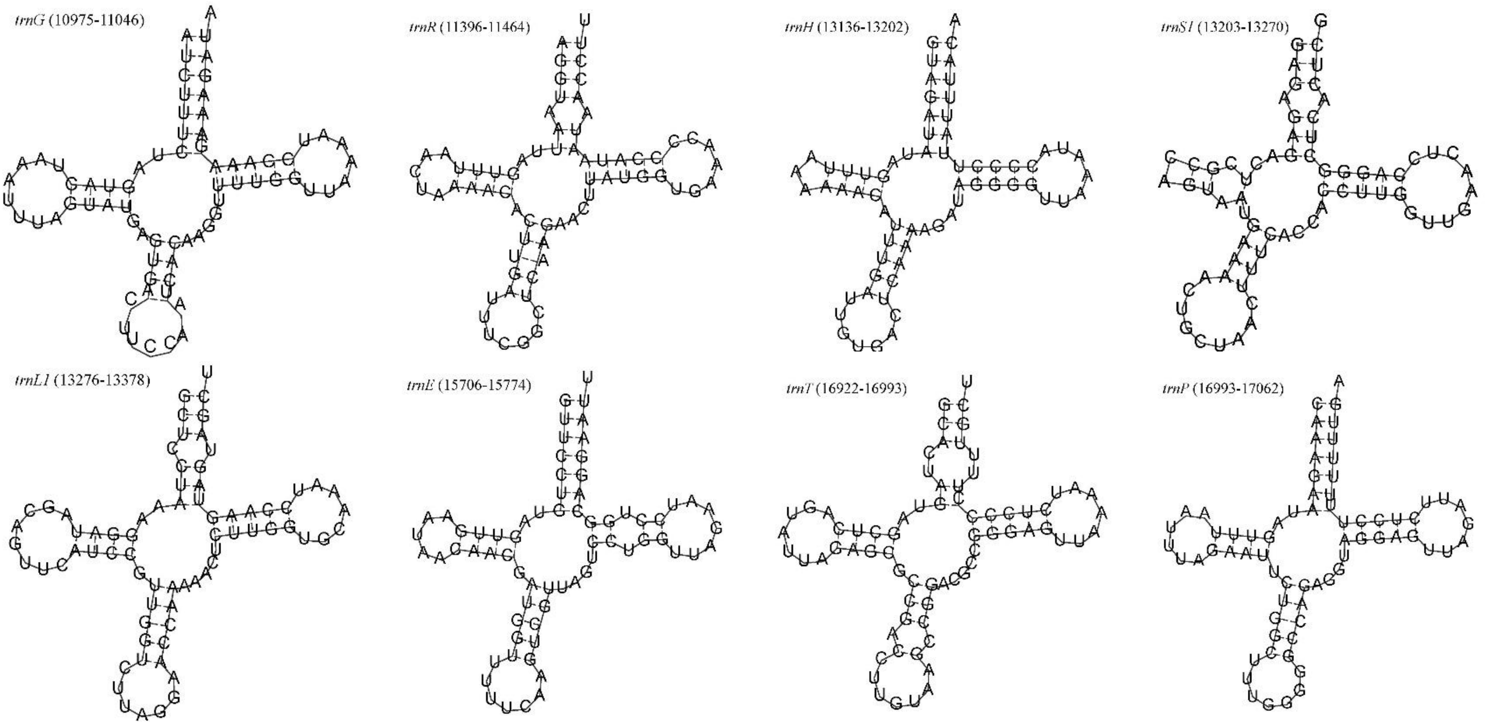
trnG (10975-11046)
trnR (11396-11464)
trnH (13136-13202)
trnS1 (13203-13270)
trnL1 (13276-13378)
trnE (15706-15774)
trnT (16922-16993)
trnP (16993-17062)

Pampus liuorum

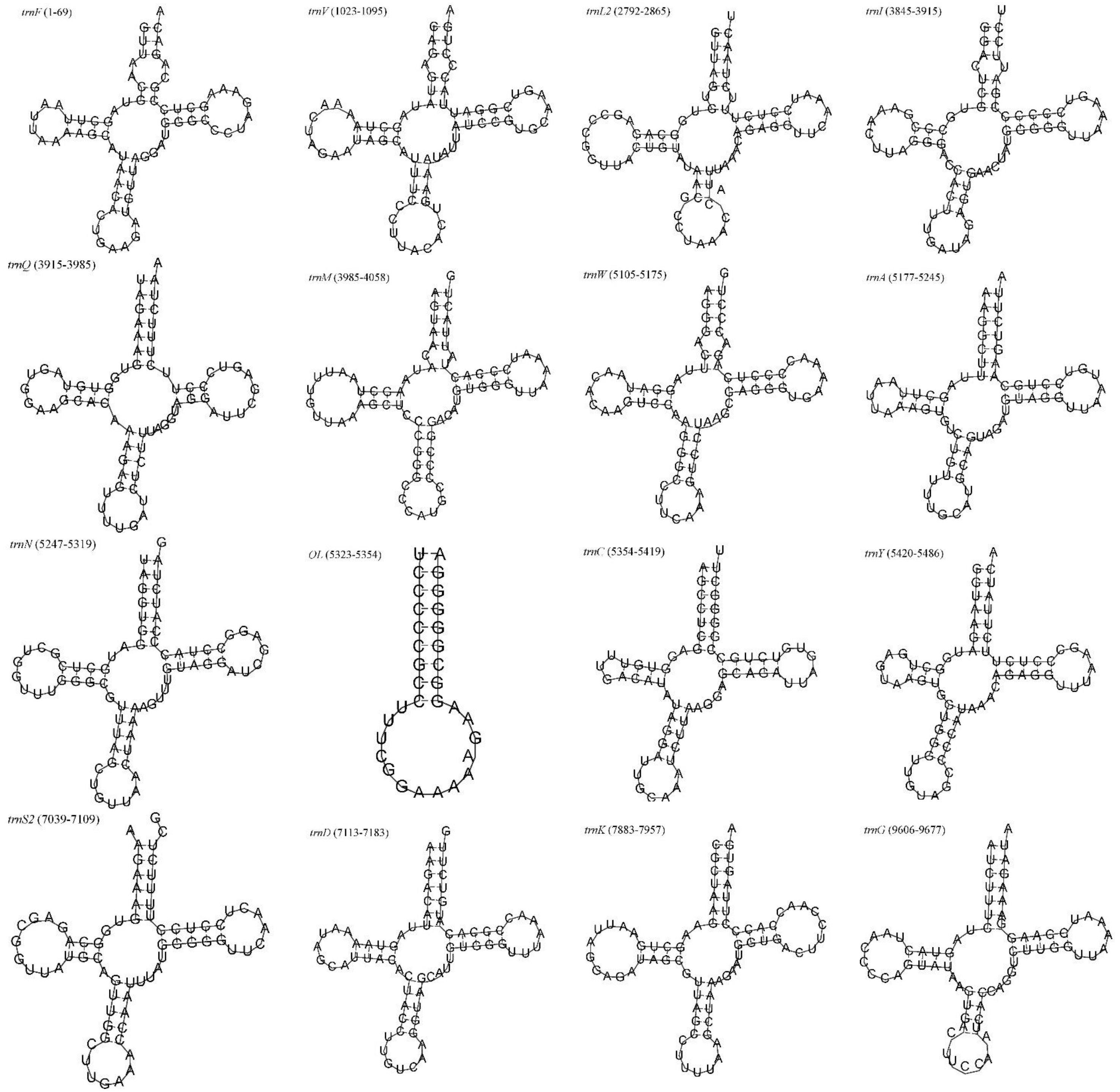

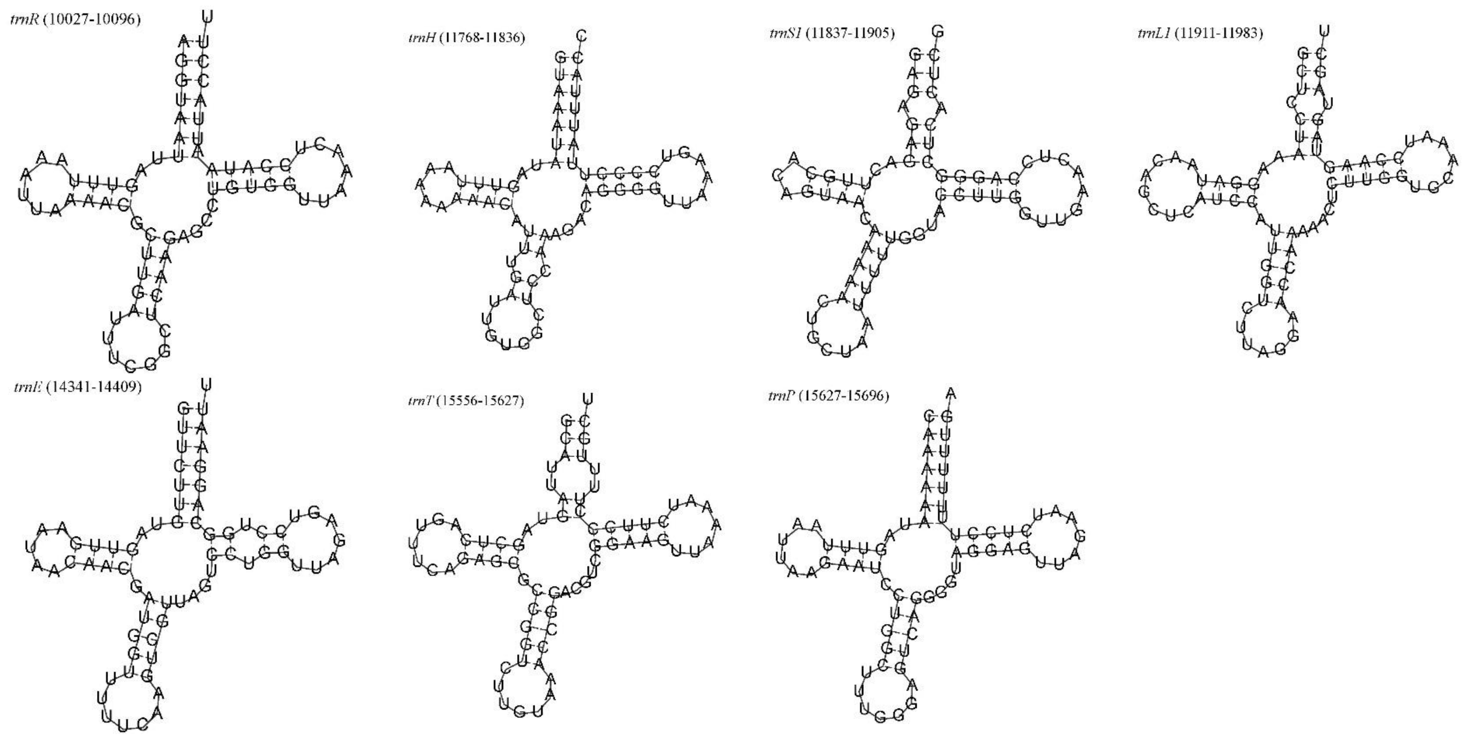

Fig. S13 Schematic diagram of the secondary structure of all tRNAs in the mitochondrial genome of the genus *Pampus*
