# Supplementary material for: Comparative Analysis of the Systematics and Evolution of the Pampus Genus of Fish (Perciformes: Stromateidae) Based on Osteology, Population Genetics and Complete Mitogenomes
Source: Animals (Basel). 2024 Mar 6;14(5):814. doi: 10.3390/ani14050814 (PMC10930999; doi:10.3390/ani14050814)
Supplement: Supplementary file 1 [file animals-14-00814-s001.zip › Supplementary information/Fig. S2.pdf]

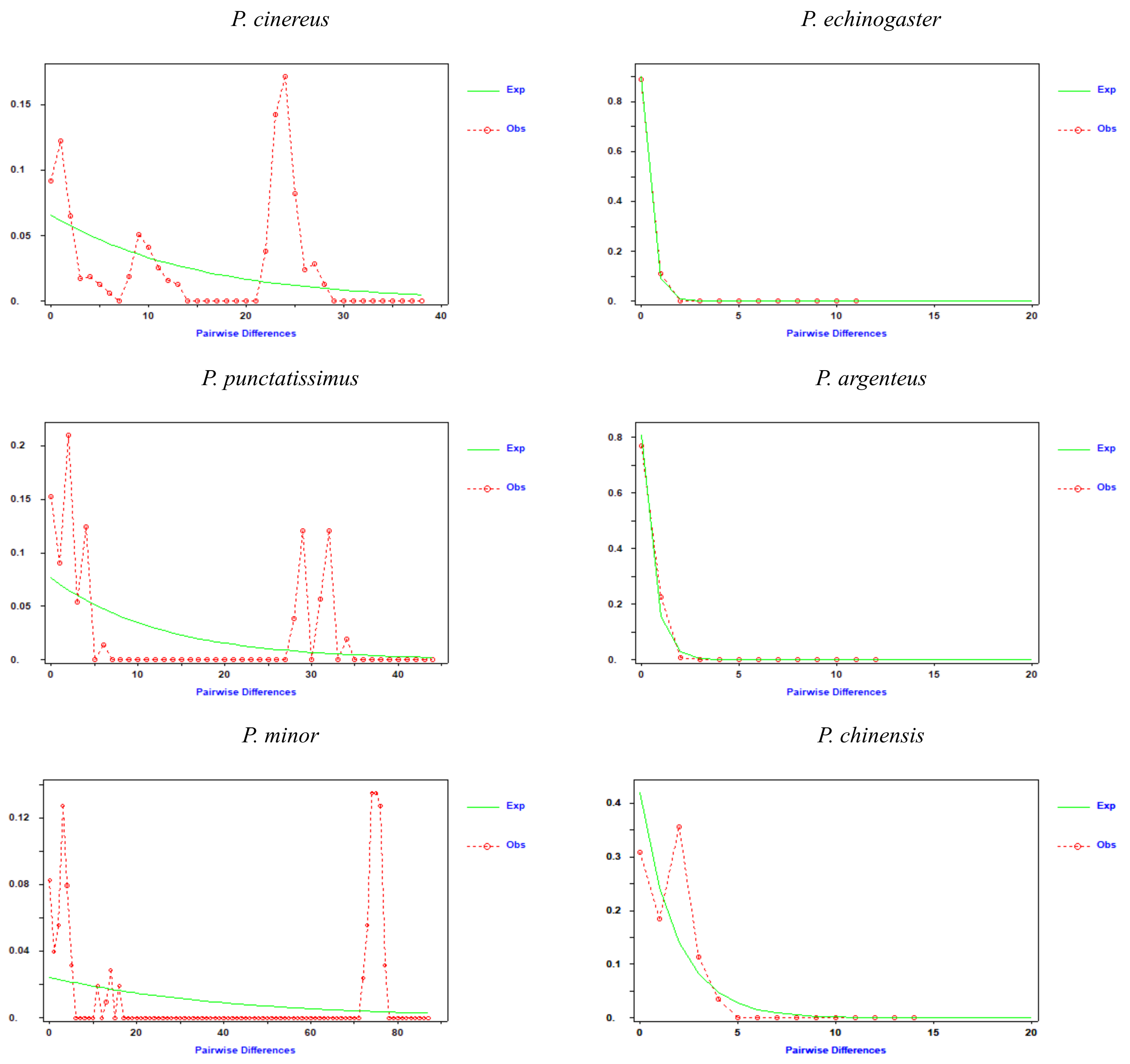

Fig. S2 Mismatch distribution for demographic expansion of the genus *Pampus* based on *D-loop* sequence
