# Supplementary material for: Comparative Analysis of the Systematics and Evolution of the Pampus Genus of Fish (Perciformes: Stromateidae) Based on Osteology, Population Genetics and Complete Mitogenomes
Source: Animals (Basel). 2024 Mar 6;14(5):814. doi: 10.3390/ani14050814 (PMC10930999; doi:10.3390/ani14050814)
Supplement: Supplementary file 1 [file animals-14-00814-s001.zip › Supplementary information/Table S1.pdf]

Table S1. Summary of complete mitochondrial gene/element features of *P. argenteus*, *P. punctatissimus*, *P. cinereus*, *P. chinensis*, *P. echinogaster*, *P. minor* and *P. liuorum*

Species	Gene/element	Strand	Position		Size (bp)	Inferred start codon	Inferred stop codon	Anti-codon	Intergenic nucleotide* (bp)
			From	To					
P. argenteus	*trnF*	+	1	69	69	–	–	GAA	0
	rrnS	+	70	1023	954	–	–	–	0
	trnV	+	1024	1096	73	–	–	TAC	26
	rrnL	+	1123	2774	1652	–	–	–	10
	trnL2	+	2785	2858	74	–	–	TAA	0
	nad1	+	2859	3833	975	ATG	TAA	–	4
	trnI	+	3838	3908	71	–	–	GAT	-1
	trnQ	-	3908	3978	71	–	–	TTG	-1
	trnM	+	3978	4051	74	–	–	CAT	23
	trnM	+	4075	4147	73	–	–	CAT	0
	nad2	+	4148	5194	1047	ATG	TAA	–	-1
	trnW	+	5194	5264	71	–	–	TCA	2
	trnA	-	5267	5335	69	–	–	TGC	1
	trnN	-	5337	5409	73	–	–	GTT	4
	O_L	+	5414	5445	32	–	–	–	-1
	trnC	-	5445	5510	66	–	–	GCA	0
	trnY	-	5511	5577	67	–	–	GTA	1
	cox1	+	5579	7132	1554	GTG	AGA	–	-5
	trnS2	-	7128	7198	71	–	–	TGA	3
	trnD	+	7202	7271	70	–	–	GTC	8
	cox2	+	7280	7970	691	ATG	T	–	0
	trnK	+	7971	8045	75	–	–	TTT	7
	atp8	+	8053	8220	168	ATG	TAA	–	-10
	atp6	+	8211	8894	684	ATG	TAA	–	-1
	cox3	+	8894	9679	786	ATG	TAA	–	-1
	trnG	+	9679	9750	72	–	–	TCC	0
	nad3	+	9751	10101	351	ATG	TAG	–	-2
	trnR	+	10100	10169	70	–	–	TCG	0
	nad4l	+	10170	10466	297	ATG	TAA	–	20
	nad4	+	10487	11840	1354	ATG	T	–	0
	trnH	+	11841	11909	69	–	–	GTG	0
	trnS1	+	11910	11978	69	–	–	GCT	5
	trnL1	+	11984	12056	73	–	–	TAG	0
	nad5	+	12057	13895	1839	ATG	TAG	–	-4
	nad6	-	13892	14413	522	GTG	TAA	–	0
	trnE	-	14414	14482	69	–	–	TTC	5
	cob	+	14488	15624	1137	ATG	TAA	–	4
	trnT	+	15629	15700	72	–	–	TGT	-1
	trnP	-	15700	15770	71	–	–	TGG	258
	OH	+	16029	16550	522	–	–	–	548

*Intergenic nucleotide: non-coding bases between the features, with a negative number indicating an overlap; – means not applicable.

Species	Gene/element	Strand	Position		Size (bp)	Inferred start codon	Inferred stop codon	Anti-codon	Intergenic nucleotide* (bp)
			From	To					
P. punctatissimus	*trnF*	+	1	69	69	–	–	GAA	0
	rrnS	+	70	1022	953	–	–	–	1
	trnV	+	1024	1096	73	–	–	TAC	25
	rrnL	+	1122	2788	1667	–	–	–	0
	trnL2	+	2789	2862	74	–	–	TAA	0
	nad1	+	2863	3837	975	ATG	TAA	–	4
	trnI	+	3842	3912	71	–	–	GAT	-1
	trnQ	-	3912	3982	71	–	–	TTG	-1
	trnM	+	3982	4055	74	–	–	CAT	0
	nad2	+	4056	5102	1047	ATG	TAA	–	-1
	trnW	+	5102	5172	71	–	–	TCA	1
	trnA	-	5174	5242	69	–	–	TGC	1
	trnN	-	5244	5316	73	–	–	GTT	3
	O_L	+	5320	5351	32	–	–	–	-1
	trnC	-	5351	5416	66	–	–	GCA	0
	trnY	-	5417	5483	67	–	–	GTA	1
	cox1	+	5485	7035	1551	GTG	TAA	–	0
	trnS2	-	7036	7106	71	–	–	TGA	3
	trnD	+	7110	7182	73	–	–	GTC	8
	cox2	+	7191	7881	691	ATG	T	–	0
	trnK	+	7882	7956	75	–	–	TTT	28
	atp8	+	7985	8152	168	ATG	TAA	–	-16
	atp6	+	8137	8826	690	CTG	TAA	–	-1
	cox3	+	8826	9611	786	ATG	TAA	–	-1
	trnG	+	9611	9682	72	–	–	TCC	0
	nad3	+	9683	10031	349	ATG	T	–	0
	trnR	+	10032	10101	70	–	–	TCG	0
	nad4l	+	10102	10398	297	ATG	TAA	–	-7
	nad4	+	10392	11772	1381	GTG	T	–	0
	trnH	+	11773	11842	70	–	–	GTG	0
	trnS1	+	11843	11911	69	–	–	GCT	5
	trnL1	+	11917	11989	73	–	–	TAG	0
	nad5	+	11990	13828	1839	ATG	TAA	–	-4
	nad6	-	13825	14334	510	ATG	TAA	–	-39
	OH	+	14296	14340	45	–	–	–	6
	trnE	-	14347	14415	69	–	–	TTC	5
	cob	+	14421	15557	1137	ATG	TAA	–	4
	trnT	+	15562	15633	72	–	–	TGT	-1
	trnP	-	15633	15702	70	–	–	TGG	277
	OH	+	15980	16498	519	–	–	–	56

*Intergenic nucleotide: non-coding bases between the features, with a negative number indicating an overlap; – means not applicable.

Species	Gene/element	Strand	Position		Size (bp)	Inferred start codon	Inferred stop codon	Anti-codon	Intergenic nucleotide* (bp)
			From	To					
	trnF	+	1	69	69	–	–	GAA	0
	rrnS	+	70	1023	954	–	–	–	1
	trnV	+	1025	1097	73	–	–	TAC	26
	rrnL	+	1124	2792	1669	–	–	–	0
	trnL2	+	2793	2866	74	–	–	TAA	0
	nad1	+	2867	3841	975	ATG	TAA	–	4
	trnI	+	3846	3916	71	–	–	GAT	-1
	trnQ	-	3916	3986	71	–	–	TTG	-1
	trnM	+	3986	4060	75	–	–	CAT	1
	nad2	+	4062	5108	1047	ATG	TAA	–	-1
	trnW	+	5108	5178	71	–	–	TCA	1
	trnA	-	5180	5248	69	–	–	TGC	1
	trnN	-	5250	5322	73	–	–	GTT	3
	O_L	+	5326	5357	32	–	–	–	-1
	trnC	-	5357	5422	66	–	–	GCA	0
	trnY	-	5423	5489	67	–	–	GTA	1
	cox1	+	5491	7041	1551	GTG	TAA	–	0
	trnS2	-	7042	7112	71	–	–	TGA	3
	trnD	+	7116	7186	71	–	–	GTC	8
P. cinereus	*cox2*	+	7195	7885	691	ATG	T	–	0
	trnK	+	7886	7960	75	–	–	TTT	22
	atp8	+	7983	8150	168	ATG	TAA	–	-16
	atp6	+	8135	8824	690	CTG	TAA	–	-1
	cox3	+	8824	9609	786	ATG	TAA	–	-1
	trnG	+	9609	9680	72	–	–	TCC	0
	nad3	+	9681	10031	351	ATG	TAG	–	-2
	trnR	+	10030	10099	70	–	–	TCG	0
	nad4l	+	10100	10396	297	ATG	TAA	–	-7
	nad4	+	10390	11770	1381	GTG	T	–	0
	trnH	+	11771	11839	69	–	–	GTG	0
	trnS1	+	11840	11908	69	–	–	GCT	5
	trnL1	+	11914	11986	73	–	–	TAG	0
	nad5	+	11987	13825	1839	ATG	TAA	–	-4
	nad6	-	13822	14331	510	ATG	TAG	–	12
	trnE	-	14344	14412	69	–	–	TTC	5
	cob	+	14418	15554	1137	ATG	TAA	–	4
	trnT	+	15559	15630	72	–	–	TGT	-1
	trnP	-	15630	15699	70	–	–	TGG	280
	OH	+	15980	16492	513	–	–	–	70

*Intergenic nucleotide: non-coding bases between the features, with a negative number indicating an overlap; – means not applicable.

Species	Gene/element	Strand	Position		Size (bp)	Inferred start codon	Inferred stop codon	Anti-codon	Intergenic nucleotide* (bp)
			From	To					
	trnF	+	1	69	69	–	–	GAA	0
	rrnS	+	70	1022	953	–	–	–	1
	trnV	+	1024	1096	73	–	–	TAC	26
	rrnL	+	1123	2790	1668	–	–	–	0
	trnL2	+	2791	2864	74	–	–	TAA	0
	nad1	+	2865	3839	975	ATG	TAA	–	4
	trnI	+	3844	3914	71	–	–	GAT	-1
	trnQ	-	3914	3984	71	–	–	TTG	-1
	trnM	+	3984	4057	74	–	–	CAT	0
	nad2	+	4058	5104	1047	ATG	TAA	–	-1
	trnW	+	5104	5174	71	–	–	TCA	1
	trnA	-	5176	5244	69	–	–	TGC	1
	trnN	-	5246	5318	73	–	–	GTT	3
	O_L	+	5322	5353	32	–	–	–	-1
	trnC	-	5353	5418	66	–	–	GCA	0
	trnY	-	5419	5485	67	–	–	GTA	1
	cox1	+	5487	7037	1551	GTG	TAA	–	0
	trnS2	-	7038	7108	71	–	–	TGA	3
	trnD	+	7112	7184	73	–	–	GTC	8
P. chinensis	*cox2*	+	7193	7883	691	ATG	T	–	0
	trnK	+	7884	7958	75	–	–	TTT	28
	atp8	+	7987	8154	168	ATG	TAA	–	50
	atp6	+	8205	8828	624	ATA	TAA	–	-1
	cox3	+	8828	9613	786	ATG	TAA	–	-1
	trnG	+	9613	9684	72	–	–	TCC	0
	nad3	+	9685	10033	349	ATG	T	–	0
	trnR	+	10034	10103	70	–	–	TCG	0
	nad4l	+	10104	10400	297	ATG	TAA	–	-7
	nad4	+	10394	11774	1381	GTG	T	–	0
	trnH	+	11775	11843	69	–	–	GTG	0
	trnS1	+	11844	11912	69	–	–	GCT	5
	trnL1	+	11918	11990	73	–	–	TAG	0
	nad5	+	11991	13829	1839	ATG	TAA	–	-4
	nad6	-	13826	14335	510	ATG	TAA	–	12
	trnE	-	14348	14416	69	–	–	TTC	5
	cob	+	14422	15558	1137	ATG	TAA	–	4
	trnT	+	15563	15634	72	–	–	TGT	-1
	trnP	-	15634	15703	70	–	–	TGG	229
	OH	+	15933	16479	547	–	–	–	56

*Intergenic nucleotide: non-coding bases between the features, with a negative number indicating an overlap; – means not applicable.

Species	Gene/element	Strand	Position		Size (bp)	Inferred start codon	Inferred stop codon	Anti-codon	Intergenic nucleotide* (bp)
			From	To					
P. echinogaster	*trnF*	+	1	69	69	–	–	GAA	0
	rrnS	+	70	1023	954	–	–	–	0
	trnV	+	1024	1096	73	–	–	TAC	26
	rrnL	+	1123	2774	1652	–	–	–	10
	trnL2	+	2785	2858	74	–	–	TAA	0
	nad1	+	2859	3833	975	ATG	TAA	–	4
	trnI	+	3838	3908	71	–	–	GAT	-1
	trnQ	-	3908	3978	71	–	–	TTG	-1
	trnM	+	3978	4051	74	–	–	CAT	23
	trnM	+	4075	4147	73	–	–	CAT	0
	nad2	+	4148	5194	1047	ATG	TAA	–	-1
	trnW	+	5194	5264	71	–	–	TCA	2
	trnA	-	5267	5335	69	–	–	TGC	1
	trnN	-	5337	5409	73	–	–	GTT	4
	O_L	+	5414	5445	32	–	–	–	-1
	trnC	-	5445	5510	66	–	–	GCA	0
	trnY	-	5511	5577	67	–	–	GTA	1
	cox1	+	5579	7132	1554	GTG	AGA	–	-5
	trnS2	-	7128	7198	71	–	–	TGA	3
	trnD	+	7202	7271	70	–	–	GTC	8
	cox2	+	7280	7970	691	ATG	T	–	0
	trnK	+	7971	8045	75	–	–	TTT	7
	atp8	+	8053	8220	168	ATG	TAA	–	-10
	atp6	+	8211	8894	684	ATG	TAA	–	-1
	cox3	+	8894	9679	786	ATG	TAA	–	-1
	trnG	+	9679	9750	72	–	–	TCC	0
	nad3	+	9751	10101	351	ATG	TAG	–	-2
	trnR	+	10100	10169	70	–	–	TCG	0
	nad4l	+	10170	10466	297	ATG	TAA	–	20
	nad4	+	10487	11840	1354	ATG	T	–	0
	trnH	+	11841	11909	69	–	–	GTG	0
	trnS1	+	11910	11978	69	–	–	GCT	5
	trnL1	+	11984	12056	73	–	–	TAG	0
	nad5	+	12057	13895	1839	ATG	TAG	–	-4
	nad6	-	13892	14413	522	GTG	TAA	–	0
	trnE	-	14414	14482	69	–	–	TTC	5
	cob	+	14488	15624	1137	ATG	TAA	–	4
	trnT	+	15629	15700	72	–	–	TGT	-1
	trnP	-	15700	15770	71	–	–	TGG	258
	OH	+	16029	16550	522	–	–	–	1144

*Intergenic nucleotide: non-coding bases between the features, with a negative number indicating an overlap; – means not applicable.

Species	Gene/element	Strand	Position		Size (bp)	Inferred start codon	Inferred stop codon	Anti-codon	Intergenic nucleotide* (bp)
			From	To					
P. minor	*trnF*	+	1	69	69	–	–	GAA	0
	rrnS	+	70	1024	955	–	–	–	2
	trnV	+	1027	1099	73	–	–	TAC	25
	rrnL	+	1125	2786	1662	–	–	–	98
	nad1	+	2885	3835	951	ATT	TAA	–	46
	trnP	-	3882	3949	68	–	–	TGG	264
	OH	+	4214	4592	379	–	–	–	399
	trnL2	+	4992	5066	75	–	–	TAA	77
	OH	+	5144	5209	66	–	–	–	0
	trnI	+	5210	5280	71	–	–	GAT	-1
	trnQ	-	5280	5350	71	–	–	TTG	-1
	trnM	+	5350	5424	75	–	–	CAT	0
	nad2	+	5425	6471	1047	ATG	TAA	–	-1
	trnW	+	6471	6541	71	–	–	TCA	2
	trnA	-	6544	6612	69	–	–	TGC	1
	trnN	-	6614	6686	73	–	–	GTT	4
	O_L	+	6691	6722	32	–	–	–	-1
	trnC	-	6722	6787	66	–	–	GCA	0
	trnY	-	6788	6854	67	–	–	GTA	1
	cox1	+	6856	8406	1551	GTG	TAA	–	0
	trnS2	-	8407	8477	71	–	–	TGA	3
	trnD	+	8481	8553	73	–	–	GTC	8
	cox2	+	8562	9252	691	ATG	T	–	0
	trnK	+	9253	9327	75	–	–	TTT	21
	atp8	+	9349	9516	168	ATG	TAA	–	-10
	atp6	+	9507	10190	684	ATG	TAA	–	-1
	cox3	+	10190	10975	786	ATG	TAA	–	-1
	trnG	+	10975	11046	72	–	–	TCC	0
	nad3	+	11047	11397	351	ATG	TAG	–	-2
	trnR	+	11396	11464	69	–	–	TCG	0
	nad4l	+	11465	11761	297	ATG	TAA	–	-7
	nad4	+	11755	13135	1381	ATG	T	–	0
	trnH	+	13136	13202	67	–	–	GTG	0
	trnS1	+	13203	13270	68	–	–	GCT	5
	trnL1	+	13276	13348	73	–	–	TAG	0
	nad5	+	13349	15187	1839	ATG	TAA	–	-4
	nad6	-	15184	15705	522	GTG	TAG	–	0
	trnE	-	15706	15774	69	–	–	TTC	5
	cob	+	15780	16916	1137	ATG	TAG	–	5
	trnT	+	16922	16993	72	–	–	TGT	-1
	trnP	-	16993	17062	70	–	–	TGG	264
	OH	+	17327	17705	379	–	–	–	358

*Intergenic nucleotide: non-coding bases between the features, with a negative number indicating an overlap; – means not applicable.

Species	Gene/element	Strand	Position		Size (bp)	Inferred start codon	Inferred stop codon	Anti-codon	Intergenic nucleotide* (bp)
			From	To					
	trnF	+	1	69	69	–	–	GAA	0
	rrnS	+	70	1021	952	–	–	–	1
	trnV	+	1023	1095	73	–	–	TAC	25
	rrnL	+	1121	2791	1671	–	–	–	0
	trnL2	+	2792	2865	74	–	–	TAA	0
	nad1	+	2866	3840	975	ATG	TAA	–	4
	trnI	+	3845	3915	71	–	–	GAT	-1
	trnQ	-	3915	3985	71	–	–	TTG	-1
	trnM	+	3985	4058	74	–	–	CAT	0
	nad2	+	4059	5105	1047	ATG	TAA	–	-1
	trnW	+	5105	5175	71	–	–	TCA	1
	trnA	-	5177	5245	69	–	–	TGC	1
	trnN	-	5247	5319	73	–	–	GTT	3
	O_L	+	5323	5354	32	–	–	–	-1
	trnC	-	5354	5419	66	–	–	GCA	0
	trnY	-	5420	5486	67	–	–	GTA	1
	cox1	+	5488	7038	1551	GTG	TAA	–	0
	trnS2	-	7039	7109	71	–	–	TGA	3
	trnD	+	7113	7183	71	–	–	GTC	8
P. liuorum	*cox2*	+	7192	7882	691	ATG	T	–	0
	trnK	+	7883	7957	75	–	–	TTT	22
	atp8	+	7980	8147	168	ATG	TAA	–	-10
	atp6	+	8138	8821	684	ATG	TAA	–	-1
	cox3	+	8821	9606	786	ATG	TAA	–	-1
	trnG	+	9606	9677	72	–	–	TCC	0
	nad3	+	9678	10028	351	ATG	TAG	–	-2
	trnR	+	10027	10096	70	–	–	TCG	0
	nad4l	+	10097	10393	297	ATG	TAA	–	-7
	nad4	+	10387	11767	1381	GTG	T	–	0
	trnH	+	11768	11836	69	–	–	GTG	0
	trnS1	+	11837	11905	69	–	–	GCT	5
	trnL1	+	11911	11983	73	–	–	TAG	0
	nad5	+	11984	13822	1839	ATG	TAA	–	-4
	nad6	-	13819	14328	510	ATG	TAG	–	12
	trnE	-	14341	14409	69	–	–	TTC	5
	cob	+	14415	15551	1137	ATG	TAA	–	4
	trnT	+	15556	15627	72	–	–	TGT	-1
	trnP	-	15627	15696	70	–	–	TGG	270
	OH	+	15967	16487	521	–	–	–	55

*Intergenic nucleotide: non-coding bases between the features, with a negative number indicating an overlap; – means not applicable.
