# Supplementary material for: Comparative Analysis of the Systematics and Evolution of the Pampus Genus of Fish (Perciformes: Stromateidae) Based on Osteology, Population Genetics and Complete Mitogenomes
Source: Animals (Basel). 2024 Mar 6;14(5):814. doi: 10.3390/ani14050814 (PMC10930999; doi:10.3390/ani14050814)
Supplement: Supplementary file 1 [file animals-14-00814-s001.zip › Supplementary information/Table S2.pdf]

Table S2. Description statistics of the codon number and relative synonymous codon usage (RSCU) in 13 protein coding genes of the genus *Pampus*

Species	Codon	Count	RSCU	Codon	Count	RSCU	Codon	Count	RSCU	Codon	Count	RSCU
	UUU (F)	8.7	0.94	UCU (S)	3.6	1.18	UAU (Y)	3.5	0.8	UGU (C)	0.8	0.81
	UUC (F)	9.8	1.06	UCC (S)	4.7	1.54	UAC (Y)	5.2	1.2	UGC (C)	1.2	1.19
	UUA (L)	9.5	1.15	UCA (S)	6.2	2.04	UAA (*)	0.7	0.22	UGA (*)	8.5	2.73
	UUG (L)	1.7	0.2	UCG (S)	0.2	0.05	UAG (*)	0.2	0.05	UGG (W)	0.5	1
	CUU (L)	10.5	1.27	CCU (P)	4.7	1.13	CAU (H)	3.2	0.79	CGU (R)	0.9	0.99
	CUC (L)	10.2	1.23	CCC (P)	5.5	1.33	CAC (H)	5	1.21	CGC (R)	0.8	0.9
	CUA (L)	15.7	1.89	CCA (P)	6	1.44	CAA (Q)	6.6	1.83	CGA (R)	3.7	3.95
P. cinereus	CUG (L)	2.2	0.26	CCG (P)	0.4	0.09	CAG (Q)	0.6	0.17	CGG (R)	0.2	0.16
	AUU (I)	13.7	1.29	ACU (T)	5.8	0.95	AAU (N)	3	0.59	AGU (S)	1.1	0.35
	AUC (I)	9.3	0.88	ACC (T)	8.2	1.33	AAC (N)	7.2	1.41	AGC (S)	2.5	0.83
	AUA (I)	8.8	0.83	ACA (T)	10.2	1.64	AAA (K)	5.8	1.97	AGA (R)	0	0
	AUG (M)	3.7	1	ACG (T)	0.5	0.07	AAG (K)	0.1	0.03	AGG (R)	0	0
	GUU (V)	5.1	1.31	GCU (A)	5.8	0.94	GAU (D)	2.3	0.83	GGU (G)	3.1	0.69
	GUC (V)	2.4	0.61	GCC (A)	9.7	1.57	GAC (D)	3.2	1.17	GGC (G)	5.6	1.25
	GUA (V)	6.8	1.74	GCA (A)	8.5	1.38	GAA (E)	6.7	1.69	GGA (G)	7.3	1.63
	GUG (V)	1.3	0.34	GCG (A)	0.7	0.11	GAG (E)	1.2	0.31	GGG (G)	1.9	0.43
	UUU (F)	9.2	0.96	UCU (S)	4.4	1.38	UAU (Y)	4.3	1	UGU (C)	0.5	0.58
	UUC (F)	9.9	1.04	UCC (S)	3.8	1.19	UAC (Y)	4.3	1	UGC (C)	1.3	1.42
	UUA (L)	10.8	1.34	UCA (S)	6.4	2.01	UAA (*)	0.6	0.21	UGA (*)	8	2.74
	UUG (L)	2	0.25	UCG (S)	0.5	0.17	UAG (*)	0.2	0.05	UGG (W)	1	1
	CUU (L)	11.5	1.43	CCU (P)	4.9	1.22	CAU (H)	2.9	0.72	CGU (R)	0.8	0.88
	CUC (L)	6.1	0.75	CCC (P)	4.9	1.22	CAC (H)	5.2	1.28	CGC (R)	0.5	0.48
	CUA (L)	15.3	1.9	CCA (P)	5.5	1.36	CAA (Q)	6.4	1.71	CGA (R)	3.8	3.92
P. echinogaster	CUG (L)	2.7	0.33	CCG (P)	0.8	0.19	CAG (Q)	1.1	0.29	CGG (R)	0.6	0.64
	AUU (I)	16.4	1.49	ACU (T)	5.1	0.87	AAU (N)	5.2	0.96	AGU (S)	1.2	0.36
	AUC (I)	6.8	0.62	ACC (T)	8.3	1.42	AAC (N)	5.6	1.04	AGC (S)	2.8	0.9
	AUA (I)	9.8	0.89	ACA (T)	9.5	1.62	AAA (K)	5.3	1.86	AGA (R)	0.1	0.08
	AUG (M)	3.5	1	ACG (T)	0.5	0.09	AAG (K)	0.4	0.14	AGG (R)	0	0
	GUU (V)	5.2	1.24	GCU (A)	6.2	1.02	GAU (D)	2	0.71	GGU (G)	3.5	0.8
	GUC (V)	2.7	0.65	GCC (A)	8.7	1.43	GAC (D)	3.6	1.29	GGC (G)	4.8	1.11
	GUA (V)	7.4	1.78	GCA (A)	8.8	1.44	GAA (E)	6.6	1.7	GGA (G)	5.8	1.34
	GUG (V)	1.4	0.33	GCG (A)	0.7	0.11	GAG (E)	1.2	0.3	GGG (G)	3.2	0.75
	UUU (F)	8.2	0.87	UCU (S)	3.8	1.26	UAU (Y)	4.2	0.98	UGU (C)	0.8	0.77
	UUC (F)	10.6	1.13	UCC (S)	4.5	1.52	UAC (Y)	4.3	1.02	UGC (C)	1.2	1.23
	UUA (L)	9.9	1.21	UCA (S)	5.7	1.91	UAA (*)	0.8	0.26	UGA (*)	8.2	2.74
	UUG (L)	1	0.12	UCG (S)	0.2	0.05	UAG (*)	0	0	UGG (W)	0.9	1
	CUU (L)	11.4	1.38	CCU (P)	4.3	1.03	CAU (H)	2.8	0.69	CGU (R)	0.6	0.66
	CUC (L)	9.2	1.11	CCC (P)	5.9	1.42	CAC (H)	5.4	1.31	CGC (R)	1	1.07
	CUA (L)	15.9	1.93	CCA (P)	6.1	1.46	CAA (Q)	6.8	1.87	CGA (R)	3.8	4.03
P. punctatissimus	CUG (L)	2	0.24	CCG (P)	0.4	0.09	CAG (Q)	0.5	0.13	CGG (R)	0.2	0.25
	AUU (I)	14.4	1.35	ACU (T)	4.3	0.69	AAU (N)	2.5	0.51	AGU (S)	1.2	0.41
	AUC (I)	8.2	0.77	ACC (T)	9.8	1.59	AAC (N)	7.4	1.49	AGC (S)	2.5	0.85
	AUA (I)	9.4	0.88	ACA (T)	10.4	1.67	AAA (K)	5.8	1.92	AGA (R)	0	0
	AUG (M)	2.8	1	ACG (T)	0.3	0.05	AAG (K)	0.2	0.08	AGG (R)	0	0
	GUU (V)	4.8	1.19	GCU (A)	5.9	0.95	GAU (D)	1.6	0.58	GGU (G)	4.2	0.92
	GUC (V)	3.1	0.76	GCC (A)	9.7	1.55	GAC (D)	4	1.42	GGC (G)	4.6	1.02
	GUA (V)	6.7	1.65	GCA (A)	8.8	1.42	GAA (E)	6.8	1.71	GGA (G)	6.5	1.45
	GUG (V)	1.6	0.4	GCG (A)	0.5	0.09	GAG (E)	1.2	0.29	GGG (G)	2.8	0.61
	UUU (F)	8	0.86	UCU (S)	3.9	1.28	UAU (Y)	3.8	0.91	UGU (C)	0.9	0.86
	UUC (F)	10.7	1.14	UCC (S)	4.6	1.51	UAC (Y)	4.6	1.09	UGC (C)	1.2	1.14
	UUA (L)	8.7	1.05	UCA (S)	5.7	1.86	UAA (*)	0.7	0.23	UGA (*)	8.3	2.72
	UUG (L)	1.7	0.2	UCG (S)	0.4	0.13	UAG (*)	0.2	0.05	UGG (W)	0.7	1
	CUU (L)	10.8	1.3	CCU (P)	4.5	1.07	CAU (H)	3.1	0.75	CGU (R)	0.8	0.82
	CUC (L)	9.8	1.18	CCC (P)	5.5	1.33	CAC (H)	5.2	1.25	CGC (R)	1	1.07
	CUA (L)	16.5	1.98	CCA (P)	6.2	1.48	CAA (Q)	6.3	1.76	CGA (R)	3.5	3.78
P. liuorum	CUG (L)	2.4	0.29	CCG (P)	0.5	0.11	CAG (Q)	0.8	0.24	CGG (R)	0.3	0.33
	AUU (I)	13.5	1.28	ACU (T)	5.8	0.95	AAU (N)	3.1	0.62	AGU (S)	1	0.33
	AUC (I)	9.1	0.86	ACC (T)	8.2	1.34	AAC (N)	6.9	1.38	AGC (S)	2.8	0.9
	AUA (I)	9.1	0.86	ACA (T)	10	1.64	AAA (K)	5.8	1.95	AGA (R)	0	0
	AUG (M)	3.2	1	ACG (T)	0.5	0.08	AAG (K)	0.2	0.05	AGG (R)	0	0
	GUU (V)	4.8	1.22	GCU (A)	6.1	0.97	GAU (D)	2.2	0.81	GGU (G)	3.1	0.68
	GUC (V)	2.9	0.73	GCC (A)	9.6	1.53	GAC (D)	3.3	1.19	GGC (G)	5.6	1.25
	GUA (V)	6.5	1.64	GCA (A)	8.9	1.42	GAA (E)	6.8	1.7	GGA (G)	7.1	1.57
	GUG (V)	1.6	0.41	GCG (A)	0.5	0.09	GAG (E)	1.2	0.3	GGG (G)	2.2	0.5
	UUU (F)	9.3	0.97	UCU (S)	4.4	1.38	UAU (Y)	4.2	0.98	UGU (C)	0.5	0.58

P. argenteus	UUC (F)	9.8	1.03	UCC (S)	3.8	1.19	UAC (Y)	4.4	1.02	UGC (C)	1.3	1.42
	UUA (L)	10.8	1.34	UCA (S)	6.4	2.01	UAA (*)	0.6	0.21	UGA (*)	8.2	2.74
	UUG (L)	2	0.25	UCG (S)	0.5	0.17	UAG (*)	0.2	0.05	UGG (W)	0.8	1
	CUU (L)	11.5	1.43	CCU (P)	5	1.24	CAU (H)	2.9	0.72	CGU (R)	0.8	0.88
	CUC (L)	6.2	0.76	CCC (P)	4.8	1.21	CAC (H)	5.2	1.28	CGC (R)	0.5	0.48
	CUA (L)	15.2	1.88	CCA (P)	5.4	1.34	CAA (Q)	6.3	1.69	CGA (R)	3.8	3.92
	CUG (L)	2.8	0.34	CCG (P)	0.8	0.21	CAG (Q)	1.2	0.31	CGG (R)	0.6	0.64
	AUU (I)	16.5	1.5	ACU (T)	5.1	0.87	AAU (N)	5.1	0.94	AGU (S)	1.2	0.36
	AUC (I)	6.8	0.62	ACC (T)	8.3	1.42	AAC (N)	5.7	1.06	AGC (S)	2.8	0.9
	AUA (I)	9.8	0.89	ACA (T)	9.5	1.62	AAA (K)	5.3	1.86	AGA (R)	0.1	0.08
	AUG (M)	3.5	1	ACG (T)	0.5	0.09	AAG (K)	0.4	0.14	AGG (R)	0	0
	GUU (V)	5.2	1.24	GCU (A)	6.2	1.01	GAU (D)	2	0.71	GGU (G)	3.5	0.82
	GUC (V)	2.6	0.63	GCC (A)	8.8	1.44	GAC (D)	3.6	1.29	GGC (G)	4.7	1.09
	GUA (V)	7.5	1.81	GCA (A)	8.8	1.44	GAA (E)	6.8	1.74	GGA (G)	5.9	1.38
	GUG (V)	1.3	0.31	GCG (A)	0.7	0.11	GAG (E)	1	0.26	GGG (G)	3.1	0.71
	UUU (F)	10.2	1.1	UCU (S)	4.6	1.41	UAU (Y)	6.5	1.27	UGU (C)	1	1.04
	UUC (F)	8.3	0.9	UCC (S)	4.7	1.43	UAC (Y)	3.7	0.73	UGC (C)	0.9	0.96
	UUA (L)	13.3	1.67	UCA (S)	6.2	1.88	UAA (*)	0.6	0.22	UGA (*)	7.7	2.7
	UUG (L)	1.5	0.19	UCG (S)	0.3	0.09	UAG (*)	0.2	0.08	UGG (W)	1	1
	CUU (L)	11.8	1.48	CCU (P)	5.4	1.33	CAU (H)	4.3	1.12	CGU (R)	0.8	0.86
	CUC (L)	5.6	0.71	CCC (P)	4.3	1.07	CAC (H)	3.4	0.88	CGC (R)	0.7	0.77
	CUA (L)	13.9	1.75	CCA (P)	6.2	1.52	CAA (Q)	6.8	1.78	CGA (R)	3.6	4.03
	CUG (L)	1.6	0.2	CCG (P)	0.3	0.08	CAG (Q)	0.8	0.22	CGG (R)	0.3	0.34
P. minor	AUU (I)	17.4	1.51	ACU (T)	5.8	0.98	AAU (N)	5.9	1.14	AGU (S)	1.2	0.38
	AUC (I)	5.8	0.5	ACC (T)	7.3	1.24	AAC (N)	4.5	0.86	AGC (S)	2.7	0.82
	AUA (I)	11.5	0.99	ACA (T)	9.8	1.67	AAA (K)	6.1	1.86	AGA (R)	0	0
	AUG (M)	3.1	1	ACG (T)	0.6	0.1	AAG (K)	0.5	0.14	AGG (R)	0	0
	GUU (V)	5	1.26	GCU (A)	6.5	1.11	GAU (D)	2.7	1.06	GGU (G)	3.6	0.81
	GUC (V)	2.2	0.54	GCC (A)	7.9	1.35	GAC (D)	2.4	0.94	GGC (G)	4.2	0.95
	GUA (V)	7.2	1.8	GCA (A)	8.8	1.49	GAA (E)	6.5	1.75	GGA (G)	7.2	1.62
	GUG (V)	1.6	0.41	GCG (A)	0.3	0.05	GAG (E)	0.9	0.25	GGG (G)	2.8	0.62
	UUU (F)	7.8	0.84	UCU (S)	2.7	0.9	UAU (Y)	3.8	0.91	UGU (C)	0.8	0.77
	UUC (F)	10.8	1.16	UCC (S)	5.9	1.97	UAC (Y)	4.5	1.09	UGC (C)	1.2	1.23
	UUA (L)	9.4	1.15	UCA (S)	5.4	1.79	UAA (*)	0.8	0.27	UGA (*)	7.9	2.73
	UUG (L)	1.6	0.2	UCG (S)	0.5	0.15	UAG (*)	0	0	UGG (W)	1.2	1
	CUU (L)	10.5	1.29	CCU (P)	3.6	0.87	CAU (H)	2.6	0.62	CGU (R)	0.7	0.74
	CUC (L)	10.2	1.25	CCC (P)	6.7	1.62	CAC (H)	5.8	1.38	CGC (R)	0.9	0.99
	CUA (L)	15.3	1.88	CCA (P)	5.5	1.34	CAA (Q)	6.8	1.93	CGA (R)	3.7	3.95
	CUG (L)	1.9	0.24	CCG (P)	0.7	0.17	CAG (Q)	0.2	0.07	CGG (R)	0.3	0.33
P. chinensis	AUU (I)	13.9	1.31	ACU (T)	4.2	0.69	AAU (N)	2.3	0.47	AGU (S)	1.2	0.38
	AUC (I)	8.7	0.82	ACC (T)	9.7	1.59	AAC (N)	7.6	1.53	AGC (S)	2.4	0.79
	AUA (I)	9.3	0.87	ACA (T)	10.1	1.65	AAA (K)	5.6	1.87	AGA (R)	0	0
	AUG (M)	2.8	1	ACG (T)	0.4	0.06	AAG (K)	0.4	0.13	AGG (R)	0	0
	GUU (V)	4.8	1.18	GCU (A)	5.5	0.87	GAU (D)	1.7	0.6	GGU (G)	4.5	0.99
	GUC (V)	3.4	0.83	GCC (A)	10.3	1.65	GAC (D)	3.9	1.4	GGC (G)	4.7	1.04
	GUA (V)	5.9	1.46	GCA (A)	8.5	1.35	GAA (E)	6.9	1.75	GGA (G)	6.1	1.35
	GUG (V)	2.2	0.53	GCG (A)	0.8	0.12	GAG (E)	1	0.25	GGG (G)	2.8	0.62
