# Supplementary material for: Comparative Analysis of the Systematics and Evolution of the Pampus Genus of Fish (Perciformes: Stromateidae) Based on Osteology, Population Genetics and Complete Mitogenomes
Source: Animals (Basel). 2024 Mar 6;14(5):814. doi: 10.3390/ani14050814 (PMC10930999; doi:10.3390/ani14050814)
Supplement: Supplementary file 1 [file animals-14-00814-s001.zip › Supplementary information/Table S3.pdf]

Table S3. The optimal codon analysis of the 13 protein coding genes of the genus *Pampus*

Species	Amcide	Codon	high-expression gene (*atp6*)		low-expression gene (*nad6*)		ΔRSCU
			Number	RSCU	Number	RSCU	
P. cinereus	Phe	UUU	8	1.23	9	1.8	-0.57
		UUC	5	0.77	1	0.2	0.57
	Leu	UUA	13	1.47	13	2.69	-1.22
		UUG	4	0.45	10	2.07	-1.62
		CUU	8	0.91	4	0.83	0.08
		CUC*	10	1.13	0	0	1.13
		CUA*	12	1.36	1	0.21	1.15
		CUG	6	0.68	1	0.21	0.47
	Ile	AUU	14	1.68	5	1.67	0.01
		AUC	6	0.72	1	0.33	0.39
		AUA	5	0.6	3	1	-0.4
	Met	AUG	1	1	6	1	0
	Val	GUU	6	1.6	13	2.08	-0.48
		GUC	2	0.53	0	0	0.53
		GUA*	7	1.87	6	0.96	0.91
		GUG	0	0	6	0.96	-0.96
	Ser	UCU	0	0	6	3.27	-3.27
		UCC*	2	1.33	0	0	1.33
		UCA	2	1.33	2	1.09	0.24
		UCG	0	0	0	0	0
	Pro	CCU*	6	1.41	1	0.67	0.74
		CCC	4	0.94	1	0.67	0.27
		CCA	7	1.65	2	1.33	0.32
		CCG	0	0	2	1.33	-1.33
	Thr	ACU	5	1	3	4	-3
		ACC*	5	1	0	0	1
		ACA*	9	1.8	0	0	1.8
		ACG	1	0.2	0	0	0.2
	Ala	GCU	5	1.11	11	2.75	-1.64
		GCC*	6	1.33	0	0	1.33
		GCA*	7	1.56	1	0.25	1.31
		GCG	0	0	4	1	-1
	Tyr	UAU	2	0.67	9	2	-1.33
		UAC*	4	1.33	0	0	1.33
	TER	UAA	1	0.5	0	0	0.5
		UAG	0	0	1	1	-1
	His	CAU	2	1	0	0	1
		CAC*	2	1	0	0	1
	Gln	CAA*	6	1.5	0	0	1.5
		CAG	2	0.5	0	0	0.5
	Asn	AAU	4	0.73	0	0	0.73
		AAC*	7	1.27	2	2	-0.73
	Lys	AAA	4	2	0	0	2
		AAG	0	0	0	0	0
	Asp	GAU	0	0	3	2	-2
		GAC	0	0	0	0	0
	Glu	GAA*	6	1.71	0	0	1.71
		GAG	1	0.29	5	2	-1.71
	Cys	UGU	1	2	3	2	0
		UGC	0	0	0	0	0
	TER	UGA	5	2.5	2	2	0.5
	Trp	UGG	0	0	3	1	-1
	Arg	CGU	2	2.4	0	0	2.4
		CGC	0	0	0	0	0
		CGA	3	3.6	2	3	0.6
		CGG	0	0	2	3	-3
	Ser	AGU	3	2	3	1.64	0.36
		AGC	2	1.33	0	0	1.33
	Arg	AGA	0	0	0	0	0
		AGG	0	0	0	0	0
	Gly	GGU	1	0.57	6	1.04	-0.47
		GGC*	4	2.29	1	0.17	2.12
		GGA	2	1.14	7	1.22	-0.08
		GGG	0	0	9	1.57	-1.57

*Indicated the optimal codons

Species	Amcide	Codon	high-expression gene (*nd4l*)		low-expression gene (*nad6*)		ΔRSCU
			Number	RSCU	Number	RSCU	
P. echinogaster	Phe	UUU	2	0.57	12	2	-1.43
		UUC*	5	1.43	0	0	1.43
	Leu	UUA	1	0.26	17	3.52	-3.26
		UUG	0	0	7	1.45	-1.45
		CUU*	7	1.83	3	0.62	1.21
		CUC	2	0.52	1	0.21	0.31
		CUA*	12	3.13	1	0.21	2.92
		CUG	1	0.26	0	0	0.26
	Ile	AUU	3	1.13	7	1.91	-0.78
		AUC	0	0	0	0	0
		AUA	5	1.88	4	1.09	0.79
	Met	AUG	1	1	4	1	0
	Val	GUU	0	0	11	1.83	-1.83
		GUC	0	0	1	0.17	-0.17
		GUA	1	4	9	1.5	2.5
		GUG	0	0	3	0.5	-0.5
	Ser	UCU	2	1.09	5	2.5	-1.41
		UCC	1	0.55	1	0.5	0.05
		UCA*	6	3.27	1	0.5	2.77
		UCG	1	0.55	2	1	-0.45
	Pro	CCU	1	2	2	1.33	0.67
		CCC	0	0	0	0	0
		CCA*	1	2	1	0.67	1.33
		CCG	0	0	3	2	-2
	Thr	ACU	1	0.8	2	4	-3.2
		ACC*	4	3.2	0	0	3.2
		ACA	0	0	0	0	0
		ACG	0	0	0	0	0
	Ala	GCU	3	0.92	9	2.12	-1.2
		GCC*	5	1.54	0	0	1.54
		GCA	5	1.54	5	1.18	0.36
		GCG	0	0	3	0.71	-0.71
	Tyr	UAU	0	0	10	1.82	-1.82
		UAC	0	0	1	0.18	-0.18
	TER	UAA	1	1.5	1	0.75	0.75
		UAG	0	0	0	0	0
	His	CAU	2	1.33	1	2	-0.67
		CAC	1	0.67	0	0	0.67
	Gln	CAA	2	1.33	2	1.33	0
		CAG	1	0.67	1	0.67	0
	Asn	AAU	4	1.14	4	2	-0.86
		AAC	3	0.86	0	0	0.86
	Lys	AAA	0	0	0	0	0
		AAG	0	0	1	2	-2
	Asp	GAU	0	0	1	1	-1
		GAC	1	2	1	1	1
	Glu	GAA*	1	1	1	0.4	0.6
		GAG	1	1	4	1.6	-0.6
	Cys	UGU	1	0.67	1	2	-1.33
		UGC*	2	1.33	0	0	1.33
	TER	UGA	1	1.5	3	2.25	-0.75
	Trp	UGG	0	0	1	1	-1
	Arg	CGU	0	0	0	0	0
		CGC	1	2	0	0	2
		CGA	2	4	1	1.2	2.8
		CGG	0	0	4	4.8	-4.8
	Ser	AGU	0	0	3	1.5	-1.5
		AGC	1	0.55	0	0	0.55
	Arg	AGA	0	0	0	0	0
		AGG	0	0	0	0	0
	Gly	GGU	1	1	7	1.47	-0.47
		GGC*	2	2	1	0.21	1.79
		GGA	1	1	5	1.05	-0.05
		GGG	0	0	6	1.26	-1.26

*Indicated the optimal codons

Species	Amcide	Codon	high-expression gene (*nd4l*)		low-expression gene (*nad6*)		ΔRSCU
			Number	RSCU	Number	RSCU	
P. punctatissimus	Phe	UUU	10	1.43	3	0.75	0.68
		UUC	4	0.57	5	1.25	-0.68
	Leu	UUA*	9	1	4	0.89	0.11
		UUG	3	0.33	1	0.22	0.11
		CUU	8	0.89	5	1.11	-0.22
		CUC	6	0.67	6	1.33	-0.66
		CUA	23	2.56	9	2	0.56
		CUG	5	0.56	2	0.44	0.12
	Ile	AUU	13	1.86	8	1.71	0.15
		AUC	5	0.71	5	1.07	-0.36
		AUA	3	0.43	1	0.21	0.22
	Met	AUG	0	0	2	1	-1
	Val	GUU*	6	1.6	0	0	1.6
		GUC	3	0.8	0	0	0.8
		GUA	5	1.33	2	4	-2.67
		GUG	1	0.27	0	0	0.27
	Ser	UCU	1	0.75	2	1.71	-0.96
		UCC	0	0	1	0.86	-0.86
		UCA	2	1.5	4	3.43	-1.93
		UCG	0	0	0	0	0
	Pro	CCU*	6	1.41	2	0.89	0.52
		CCC	3	0.71	3	1.33	-0.62
		CCA	7	1.65	4	1.78	-0.13
		CCG	1	0.24	0	0	0.24
	Thr	ACU	7	1.08	1	0.5	0.58
		ACC	7	1.08	3	1.5	-0.42
		ACA	12	1.85	4	2	-0.15
		ACG	0	0	0	0	0
	Ala	GCU	6	1.2	4	2.29	-1.09
		GCC	7	1.4	2	1.14	0.26
		GCA*	5	1	1	0.57	0.43
		GCG	2	0.4	0	0	0.4
	Tyr	UAU	2	0.8	0	0	0.8
		UAC	3	1.2	2	2	-0.8
	TER	UAA	1	0.5	0	0	0.5
		UAG	0	0	0	0	0
	His	CAU	2	1	0	0	1
		CAC	2	1	1	2	-1
	Gln	CAA	9	2	2	2	0
		CAG	0	0	0	0	0
	Asn	AAU	3	0.75	0	0	0.75
		AAC	5	1.25	2	2	-0.75
	Lys	AAA	4	1.6	1	2	-0.4
		AAG	1	0.4	0	0	0.4
	Asp	GAU	1	2	0	0	2
		GAC	0	0	4	2	-2
	Glu	GAA	6	2	6	2	0
		GAG	0	0	0	0	0
	Cys	UGU	0	0	0	0	0
		UGC	0	0	1	2	-2
	TER	UGA	5	2.5	4	3	-0.5
	Trp	UGG	0	0	1	1	-1
	Arg	CGU	0	0	0	0	0
		CGC	2	3	1	3	0
		CGA	2	3	1	3	0
		CGG	0	0	0	0	0
	Ser	AGU	2	1.5	0	0	1.5
		AGC	3	2.25	0	0	2.25
	Arg	AGA	0	0	0	0	0
		AGG	0	0	0	0	0
	Gly	GGU	1	0.57	1	0.67	-0.1
		GGC	4	2.29	3	2	0.29
		GGA	2	1.14	2	1.33	-0.19
		GGG	0	0	0	0	0

*Indicated the optimal codons

Species	Amcide	Codon	high-expression gene (*atp6*)		low-expression gene (*nad6*)		ΔRSCU
			Number	RSCU	Number	RSCU	
P. liuorum	Phe	UUU	6	0.86	9	1.8	-0.94
		UUC*	8	1.14	1	0.2	0.94
	Leu	UUA	10	1.18	12	2.48	-1.3
		UUG	1	0.12	12	2.48	-2.36
		CUU*	9	1.06	4	0.83	0.23
		CUC*	9	1.06	0	0	1.06
		CUA*	15	1.76	1	0.21	1.55
		CUG	7	0.82	0	0	0.82
	Ile	AUU	13	1.56	5	1.88	-0.32
		AUC	7	0.84	0	0	0.84
		AUA	5	0.6	3	1.13	-0.53
	Met	AUG	2	1	5	1	0
	Val	GUU	6	1.6	11	1.83	-0.23
		GUC	2	0.53	0	0	0.53
		GUA	7	1.87	6	1	0.87
		GUG	0	0	7	1.17	-1.17
	Ser	UCU	0	0	6	3.27	-3.27
		UCC*	2	1.2	0	0	1.2
		UCA*	2	1.2	1	0.55	0.65
		UCG	0	0	1	0.55	-0.55
	Pro	CCU*	5	1.18	1	0.67	0.51
		CCC	4	0.94	1	0.67	0.27
		CCA	7	1.65	2	1.33	0.32
		CCG	1	0.24	2	1.33	-1.09
	Thr	ACU	5	1.05	2	2.67	-1.62
		ACC	4	0.84	0	0	0.84
		ACA*	10	2.11	0	0	2.11
		ACG	0	0	1	1.33	-1.33
	Ala	GCU	5	1.11	12	2.67	-1.56
		GCC*	6	1.33	0	0	1.33
		GCA*	6	1.33	2	0.44	0.89
		GCG	1	0.22	4	0.89	-0.67
	Tyr	UAU	2	0.8	9	2	-1.2
		UAC*	3	1.2	0	0	1.2
	TER	UAA	1	0.5	0	0	0.5
		UAG	0	0	1	1	-1
	His	CAU	1	0.5	0	0	0.5
		CAC*	3	1.5	0	0	1.5
	Gln	CAA*	6	1.5	0	0	1.5
		CAG	2	0.5	0	0	0.5
	Asn	AAU	4	0.8	0	0	0.8
		AAC	6	1.2	2	2	-0.8
	Lys	AAA*	4	2	0	0	2
		AAG	0	0	0	0	0
	Asp	GAU	0	0	3	2	-2
		GAC	0	0	0	0	0
	Glu	GAA*	6	1.71	0	0	1.71
		GAG	1	0.29	5	2	-1.71
	Cys	UGU	1	2	3	2	0
		UGC	0	0	0	0	0
	TER	UGA	5	2.5	2	2	0.5
	Trp	UGG	0	0	3	1	-1
	Arg	CGU	1	1.2	0	0	1.2
		CGC*	1	1.2	0	0	1.2
		CGA	2	2.4	3	4.5	-2.1
		CGG	1	1.2	1	1.5	-0.3
	Ser	AGU	4	2.4	3	1.64	0.76
		AGC	2	1.2	0	0	1.2
	Arg	AGA	0	0	0	0	0
		AGG	0	0	0	0	0
	Gly	GGU	1	0.57	6	1	-0.43
		GGC*	4	2.29	1	0.17	2.12
		GGA	2	1.14	7	1.17	-0.03
		GGG	0	0	10	1.67	-1.67

*Indicated the optimal codons

Species	Amcide	Codon	high-expression gene (*nad4l*)		low-expression gene (*nad6*)		ΔRSCU
			Number	RSCU	Number	RSCU	
P. argenteus	Phe	UUU	2	0.57	12	2	-1.43
		UUC*	5	1.43	0	0	1.43
	Leu	UUA	1	0.26	17	3.52	-3.26
		UUG	0	0	7	1.45	-1.45
		CUU*	7	1.83	3	0.62	1.21
		CUC	2	0.52	1	0.21	0.31
		CUA*	12	3.13	1	0.21	2.92
		CUG	1	0.26	0	0	0.26
	Ile	AUU	3	1.13	7	1.91	-0.78
		AUC	0	0	0	0	0
		AUA	5	1.88	4	1.09	0.79
	Met	AUG	1	1	4	1	0
	Val	GUU	0	0	11	1.83	-1.83
		GUC	0	0	1	0.17	-0.17
		GUA	1	4	9	1.5	2.5
		GUG	0	0	3	0.5	-0.5
	Ser	UCU	2	1.09	5	2.5	-1.41
		UCC	1	0.55	1	0.5	0.05
		UCA*	6	3.27	1	0.5	2.77
		UCG	1	0.55	2	1	-0.45
	Pro	CCU	1	2	2	1.33	0.67
		CCC	0	0	0	0	0
		CCA*	1	2	1	0.67	1.33
		CCG	0	0	3	2	-2
	Thr	ACU	1	0.8	2	4	-3.2
		ACC*	4	3.2	0	0	3.2
		ACA	0	0	0	0	0
		ACG	0	0	0	0	0
	Ala	GCU	3	0.92	8	1.88	-0.96
		GCC*	5	1.54	1	0.24	1.3
		GCA	5	1.54	5	1.18	0.36
		GCG	0	0	3	0.71	-0.71
	Tyr	UAU	0	0	10	1.82	-1.82
		UAC	0	0	1	0.18	-0.18
	TER	UAA	1	1.5	1	0.75	0.75
		UAG	0	0	0	0	0
	His	CAU	2	1.33	1	2	-0.67
		CAC	1	0.67	0	0	0.67
	Gln	CAA	2	1.33	2	1.33	0
		CAG	1	0.67	1	0.67	0
	Asn	AAU	3	0.86	4	2	-1.14
		AAC*	4	1.14	0	0	1.14
	Lys	AAA	0	0	0	0	0
		AAG	0	0	1	2	-2
	Asp	GAU	0	0	1	1	-1
		GAC	1	2	1	1	1
	Glu	GAA*	1	1	1	0.4	0.6
		GAG	1	1	4	1.6	-0.6
	Cys	UGU	1	0.67	1	2	-1.33
		UGC*	2	1.33	0	0	1.33
	TER	UGA	1	1.5	3	2.25	-0.75
	Trp	UGG	0	0	1	1	-1
	Arg	CGU	0	0	0	0	0
		CGC	1	2	0	0	2
		CGA	2	4	1	1.2	2.8
		CGG	0	0	4	4.8	-4.8
	Ser	AGU	0	0	3	1.5	-1.5
		AGC	1	0.55	0	0	0.55
	Arg	AGA	0	0	0	0	0
		AGG	0	0	0	0	0
	Gly	GGU	1	1	7	1.47	-0.47
		GGC*	2	2	1	0.21	1.79
		GGA	1	1	5	1.05	-0.05
		GGG	0	0	6	1.26	-1.26

*Indicated the optimal codons

Species	Amcide	Codon	high-expression gene (*atp6*)		low-expression gene (*nad6*)		ΔRSCU
			Number	RSCU	Number	RSCU	
P. minor	Phe	UUU	10	1.18	11	1.83	-0.65
		UUC	7	0.82	1	0.17	0.65
	Leu	UUA	17	2.17	12	2.67	-0.5
		UUG	0	0	12	2.67	-2.67
		CUU*	13	1.66	1	0.22	1.44
		CUC	1	0.13	0	0	0.13
		CUA*	12	1.53	2	0.44	1.09
		CUG	4	0.51	0	0	0.51
	Ile	AUU	19	1.73	6	1.29	0.44
		AUC	4	0.36	0	0	0.36
		AUA	10	0.91	8	1.71	-0.8
	Met	AUG	1	1	2	1	0
	Val	GUU	2	0.73	9	1.5	-0.77
		GUC	2	0.73	0	0	0.73
		GUA	6	2.18	10	1.67	0.51
		GUG	1	0.36	5	0.83	-0.47
	Ser	UCU	2	1.33	5	2.5	-1.17
		UCC*	2	1.33	0	0	1.33
		UCA	2	1.33	2	1	0.33
		UCG	0	0	1	0.5	-0.5
	Pro	CCU	5	1.25	5	3.33	-2.08
		CCC	3	0.75	0	0	0.75
		CCA*	8	2	0	0	2
		CCG	0	0	1	0.67	-0.67
	Thr	ACU	7	1.27	3	4	-2.73
		ACC*	6	1.09	0	0	1.09
		ACA*	9	1.64	0	0	1.64
		ACG	0	0	0	0	0
	Ala	GCU	4	1	11	2.59	-1.59
		GCC	2	0.5	0	0	0.5
		GCA*	10	2.5	2	0.47	2.03
		GCG	0	0	4	0.94	-0.94
	Tyr	UAU	4	1.6	9	1.64	-0.04
		UAC	1	0.4	2	0.36	0.04
	TER	UAA	1	0.75	0	0	0.75
		UAG	0	0	1	1	-1
	His	CAU*	4	2	0	0	2
		CAC	0	0	0	0	0
	Gln	CAA*	6	1.5	0	0	1.5
		CAG	2	0.5	0	0	0.5
	Asn	AAU	5	1.11	2	2	-0.89
		AAC	4	0.89	0	0	0.89
	Lys	AAA*	7	2	0	0	2
		AAG	0	0	0	0	0
	Asp	GAU	0	0	4	2	-2
		GAC	1	2	0	0	2
	Glu	GAA*	4	1.33	2	0.67	0.66
		GAG	2	0.67	4	1.33	-0.66
	Cys	UGU	0	0	2	2	-2
		UGC	0	0	0	0	0
	TER	UGA	3	2.25	2	2	0.25
	Trp	UGG	1	1	1	1	0
	Arg	CGU	1	1.5	0	0	1.5
		CGC	1	1.5	0	0	1.5
		CGA	2	3	2	3	0
		CGG	0	0	2	3	-3
	Ser	AGU	2	1.33	4	2	-0.67
		AGC	1	0.67	0	0	0.67
	Arg	AGA	0	0	0	0	0
		AGG	0	0	0	0	0
	Gly	GGU	3	1.71	8	1.33	0.38
		GGC	3	1.71	1	0.17	1.54
		GGA	1	0.57	5	0.83	-0.26
		GGG	0	0	10	1.67	-1.67

*Indicated the optimal codons

Species	Amcide	Codon	high-expression gene (*atp6*)		low-expression gene (*nad6*)		ΔRSCU
			Number	RSCU	Number	RSCU	
P. chinensis	Phe	UUU	6	1	11	2	-1
		UUC*	6	1	0	0	1
	Leu	UUA	11	1.43	11	2.36	-0.93
		UUG	3	0.39	12	2.57	-2.18
		CUU	7	0.91	4	0.86	0.05
		CUC	5	0.65	0	0	0.65
		CUA*	16	2.09	1	0.21	1.88
		CUG	4	0.52	0	0	0.52
	Ile	AUU	13	1.7	4	2.4	-0.7
		AUC	5	0.65	0	0	0.65
		AUA	5	0.65	1	0.6	0.05
	Met	AUG	0	0	7	1	-1
	Val	GUU	7	1.87	12	1.85	0.02
		GUC	2	0.53	0	0	0.53
		GUA*	4	1.07	5	0.77	0.3
		GUG	2	0.53	9	1.38	-0.85
	Ser	UCU	0	0	4	2	-2
		UCC*	1	1.2	0	0	1.2
		UCA*	2	2.4	1	0.5	1.9
		UCG	0	0	3	1.5	-1.5
	Pro	CCU	3	0.8	1	0.67	0.13
		CCC*	5	1.33	1	0.67	0.66
		CCA	7	1.87	2	1.33	0.54
		CCG	0	0	2	1.33	-1.33
	Thr	ACU	4	0.76	2	4	-3.24
		ACC*	7	1.33	0	0	1.33
		ACA*	10	1.9	0	0	1.9
		ACG	0	0	0	0	0
	Ala	GCU	5	1.05	11	2.44	-1.39
		GCC*	7	1.47	2	0.44	1.03
		GCA*	5	1.05	3	0.67	0.38
		GCG	2	0.42	2	0.44	-0.02
	Tyr	UAU	3	1.5	7	2	-0.5
		UAC	1	0.5	0	0	0.5
	TER	UAA	1	0.5	1	1	-0.5
		UAG	0	0	0	0	0
	His	CAU	3	1.5	2	2	-0.5
		CAC	1	0.5	0	0	0.5
	Gln	CAA*	8	2	0	0	2
		CAG	0	0	0	0	0
	Asn	AAU	4	1.14	0	0	1.14
		AAC	3	0.86	1	2	-1.14
	Lys	AAA*	4	1.6	0	0	1.6
		AAG	1	0.4	0	0	0.4
	Asp	GAU	1	2	3	2	0
		GAC	0	0	0	0	0
	Glu	GAA*	6	2	1	0.4	1.6
		GAG	0	0	4	1.6	-1.6
	Cys	UGU	0	0	3	2	-2
		UGC	0	0	0	0	0
	TER	UGA	5	2.5	2	2	0.5
	Trp	UGG	0	0	3	1	-1
	Arg	CGU	1	1.5	0	0	1.5
		CGC	1	1.5	0	0	1.5
		CGA	2	3	3	4.5	-1.5
		CGG	0	0	1	1.5	-1.5
	Ser	AGU	1	1.2	4	2	-0.8
		AGC	1	1.2	0	0	1.2
	Arg	AGA	0	0	0	0	0
		AGG	0	0	0	0	0
	Gly	GGU	1	0.57	7	1.17	-0.6
		GGC*	4	2.29	0	0	2.29
		GGA*	2	1.14	4	0.67	0.47
		GGG	0	0	13	2.17	-2.17

*Indicated the optimal codons.
