# Supplementary material for: Comparative Analysis of the Systematics and Evolution of the Pampus Genus of Fish (Perciformes: Stromateidae) Based on Osteology, Population Genetics and Complete Mitogenomes
Source: Animals (Basel). 2024 Mar 6;14(5):814. doi: 10.3390/ani14050814 (PMC10930999; doi:10.3390/ani14050814)
Supplement: Supplementary file 1 [file Fig. S12.pdf › Supplementary information/Fig. S12.pdf]

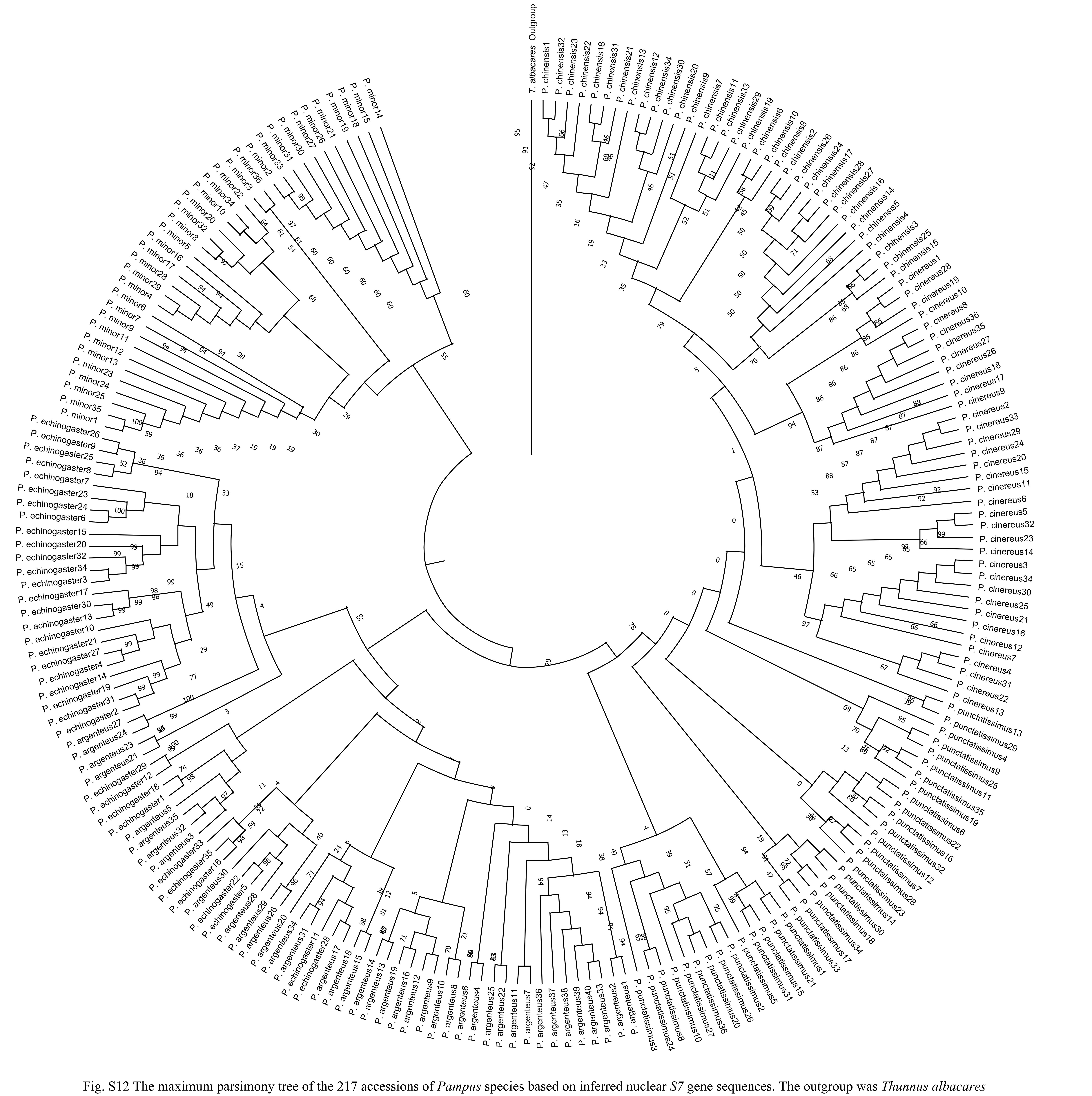


Fig. S12 The maximum parsimony tree of the 217 accessions of *Pampus* species based on inferred nuclear *S7* gene sequences. The outgroup was *Thunnus albacares*
